# Atomistic simulations of the *Escherichia coli* ribosome provide selection criteria for translationally active substrates

**DOI:** 10.1038/s41557-023-01226-w

**Published:** 2023-06-12

**Authors:** Zoe L. Watson, Isaac J. Knudson, Fred R. Ward, Scott J. Miller, Jamie H. D. Cate, Alanna Schepartz, Ara M. Abramyan

**Affiliations:** 1 Department of Chemistry, University of California, Berkeley, CA USA; 2 Center for Genetically Encoded Materials, University of California, Berkeley, CA USA; 3 California Institute for Quantitative Biosciences (QB3), University of California, Berkeley, CA USA; 4 Department of Molecular and Cellular Biology, University of California, Berkeley, CA USA; 5 Department of Chemistry, Yale University, New Haven, CT USA; 6 Molecular Biophysics and Integrated Bioimaging Division, Lawrence Berkeley National Laboratory, Berkeley, CA USA; 7 Chan Zuckerberg Biohub, San Francisco, CA USA; 8 Schrödinger, Inc., San Diego, CA USA

**Keywords:** Synthetic biology, Cryoelectron microscopy, Computational platforms and environments

## Abstract

As genetic code expansion advances beyond l-α-amino acids to backbone modifications and new polymerization chemistries, delineating what substrates the ribosome can accommodate remains a challenge. The *Escherichia coli* ribosome tolerates non-l-α-amino acids in vitro, but few structural insights that explain how are available, and the boundary conditions for efficient bond formation are so far unknown. Here we determine a high-resolution cryogenic electron microscopy structure of the *E. coli* ribosome containing α-amino acid monomers and use metadynamics simulations to define energy surface minima and understand incorporation efficiencies. Reactive monomers across diverse structural classes favour a conformational space where the aminoacyl-tRNA nucleophile is <4 Å from the peptidyl-tRNA carbonyl with a Bürgi–Dunitz angle of 76–115°. Monomers with free energy minima that fall outside this conformational space do not react efficiently. This insight should accelerate the in vivo and in vitro ribosomal synthesis of sequence-defined, non-peptide heterooligomers.

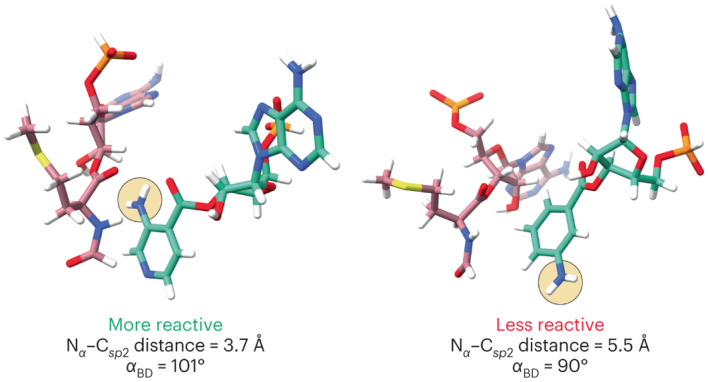

## Abstract (web summary)

Genetic code expansion to incorporate non-α-amino acid monomers is limited by predictability of monomer reactivities in the context of the ribosome. Now the use of metadynamics simulations of pre-attack monomers in the ribosomal peptidyl transferase centre provides insight on whether an A-site monomer is likely to be reactive.

## Main

The ribosome is a biological machine that faithfully converts information embodied in one polymer into a different polymer with minimal information loss, synonymous codons notwithstanding. Although the process of mRNA translation enables vast and sophisticated functions throughout biology, the protein chemical space in extant organisms is limited to about 20 natural α-amino acids and, before post-translational modifications, an invariant peptide backbone. Twenty-plus years of genetic code expansion has broadened protein chemical space to include greater than 200 non-natural α-amino acids^1^. However, with two exceptions^2,3^, protein chemical space in vivo is limited to α-amino and (a few) α-hydroxy acids and an otherwise invariant peptide backbone^4–7^.

**Fig. 1:**
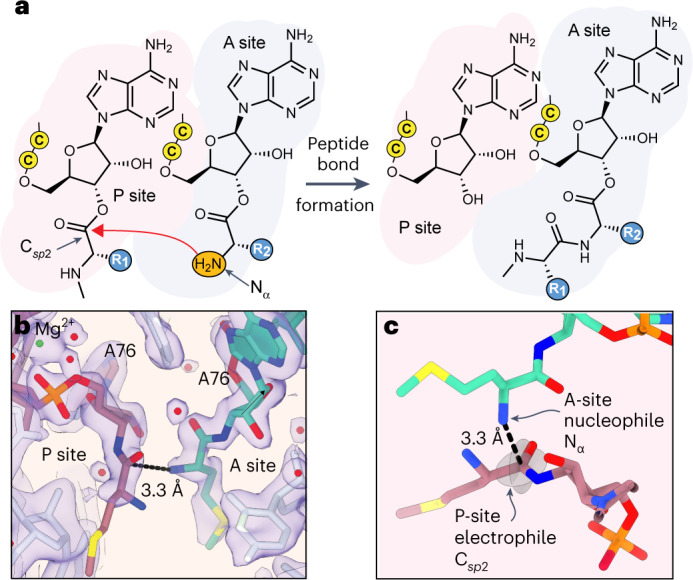
Substrate positioning in the PTC. **a**, Schematic depicting peptide bond formation in the ribosome active site known as the PTC. The PTC promotes peptide bond formation by positioning the nucleophilic α-amino group (N_α_) of one substrate, the A-site aminoacyl-tRNA, near the electrophilic *sp*^2^-hybridized carbonyl carbon (C_*sp*2_) of the second substrate, the P-site peptidyl-tRNA. Attack by N_α_ generates a tetrahedral intermediate that subsequently breaks down to product: a peptidyl-tRNA carrying an additional C-terminal amino acid. Cytidine residues of the tRNAs’ 3′-CCA ends are represented by the letter ‘C’ in a yellow circle. **b**, Cryo-EM density (indigo surface) and our model for Met residues in the PTC. The distance between the A-site (green) nucleophilic amine and the P-site (rose) carbonyl carbon (3.3 Å) is indicated with a black dotted line. The 23S rRNA is shown in white. The Mg^2+^ ion that coordinates the C75–A76 internucleotide phosphate is shown in green. Note that an amide linkage is shown between the A76 and Met residues of each tRNA to reflect the experimental substrates used for structure determination; however, for simulation, this was replaced with the ester linkage that would be present during an elongation reaction. The map here was post-processed with a B-factor of –13 Å^2^ and supersampled for smoothness. **c**, Alternative view of the A-site and P-site Met residues highlighting the planes of the carbonyl that define the Bürgi–Dunitz (*α*_BD_) and Flippin–Lodge (*α*_FL_) angles and the geometry of nucleophilic attack.

By contrast, access to alternative backbones is more widespread in vitro, where the concentration of chemically produced acyl-tRNA can be 50 times higher than possible in vivo. Under such conditions, *E. coli* ribosomes support bond-forming reactions between monomers whose backbones deviate considerably from native α-amino acids^8–15^. The structural and electronic diversity of these monomers underscores the importance of proximity in promoting bond-forming reactions within the *E. coli* peptidyl transferase centre (PTC; Fig. 1a)^16^. Yet the yields of peptides containing these unusual monomers vary widely, and the products are often detected only by use of autoradiography or mass spectrometry. Given the potential of the ribosome for novel bond-forming chemistry, one must ask: what structural features define reactive monomers? Here we describe the implementation of a structurally informed computational workflow to identify promising monomers with speed, high accuracy and low cost.

We faced three challenges in implementing such a computational schema. First, the size of the ribosome—1.5 MDa in the *E. coli* large ribosomal subunit, not including substrates and solvation—pushes computational methods to their limit^17,18^. As a result, several coarse-grained^19^, normal mode analysis^20^ and elastic network^21^ models combined with simplified ribosome representations have been developed. While these simplifications reduce computational cost and adequately describe large-scale motions, they fail to capture atomistic details. By contrast, density functional theory (DFT) can provide atomistic and electronic details but cannot yet evaluate de novo how new monomers engage the PTC in the absence of experimental constraints on orientations in the active site^22^.

The second challenge is that the ribosome is a ribozyme^23^. Most structure-based modelling tools in wide use were developed to study protein enzymes. RNA contains more degrees of freedom than protein, and the polyanionic composition demands accurate, long-range electrostatic and solvation models that include explicit metal ions and water, especially within the PTC^24^. Conformational dynamics alone remains an enormous challenge for computational analyses, with increasing challenges as the complexity of the system increases^25^. While others have used three-dimensional (3D) puzzles for RNA structural predictions^26^, physics-based methods such as molecular dynamics (MD) simulations provide a more accurate description of the atomistic dynamics of biological systems^27–29^.

**Extended Data Fig. 1:**
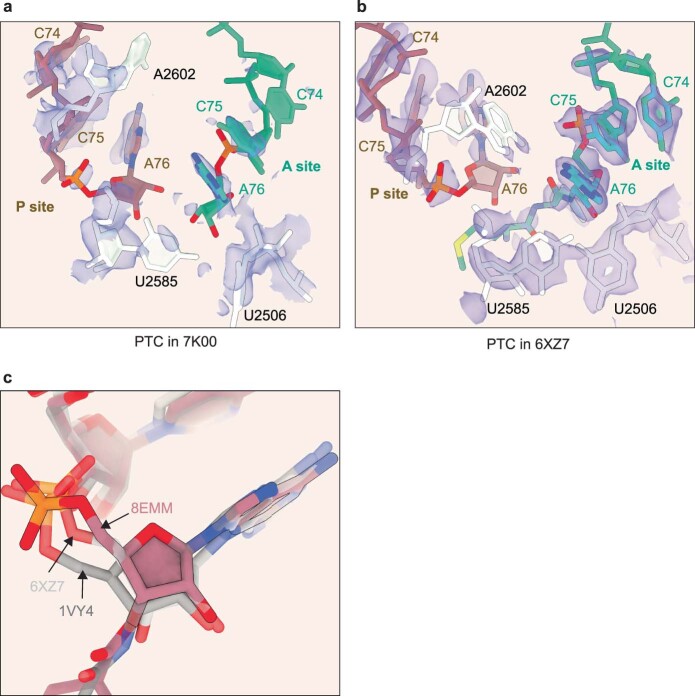
Details in the PTC of prior structures. **a**, Close-up of the PTC in PDB 7K00 illustrates the poor quality of local density in this map (indigo) around the P site (rose) and A site (green) tRNAs. PTC bases U2506, U2585, and A2602 of the 23S rRNA are shown in white. The deposited 50S-focused map is shown with an additional B-factor of 30 Å^2^ applied, supersampled for smoothness. **b**, Close-up of the PTC in PDB 6XZ7, showing dipeptide in the A site (green with atomistic coloring) and uncharged P-site tRNA (rose). The map has been supersampled for smoothness. Contour levels for maps shown in both **a** and **b** are chosen to illustrate disorder relative to better-resolved map regions, but lower contour levels and/or higher B-factor blurring brings out features supporting models. Labels for A- and P-site tRNAs in **a** and **b** are color-matched to the carbon atoms, and 23S rRNA bases are labeled in black. **c**, P-site tRNA A76 backbone conformation in our model (pink, black outline) compared to 6XZ7 (light gray) and 1VY4 (dark gray). Labels indicating the models are color-matched to the carbon atoms.

The final challenge is the choice of an appropriate high-resolution reference state. The highest-resolution structure of the ribosome available at the time of this work was Protein Data Bank (PDB) 7K00 (ref. ^30^). This cryogenic electron microscopy (cryo-EM) structure includes the 70S *E. coli* ribosome in complex with tRNA and mRNA substrates at 2 Å global resolution. Although local resolution surpassed this value in regions of the large subunit, the PTC itself was less well resolved, and the monomers could not be modelled (Extended Data Fig. 1a). PDB 6XZ7 (ref. ^31^) is also high resolution (2.1 Å) but contains product-like tRNAs (Extended Data Fig. 1b); PDB 1VY4 (ref. ^24^) contains reactant-like tRNAs, but the lower resolution (2.6 Å) as well as local map quality of amino acids in the PTC obscures details of monomer placement. We concluded that neither PDB 7K00, nor 6XZ7, nor 1VY4 represented an ideal starting point for simulations to evaluate the conformational landscape of non-l-α-amino acid monomers in the *E. coli* PTC; a structure with well-resolved monomers was required.

Here we present a cryo-EM structure of the *E. coli* ribosome that reaches 2.1 Å global resolution in the large subunit and visualizes natural methionine monomers and full-length tRNAs at improved resolution. Using a model derived from this structure, we executed metadynamics simulations to define the conformational free energy surfaces (FESs) of multiple structurally and stereochemically diverse non-α-amino acid monomers within the PTC. Minima in these FESs clearly differentiate reactive and non-reactive monomers: reactive monomers across all structural classes populate a conformational space characterized by an A-site nucleophile to P-site carbonyl distance (N_α_–C_*sp*2_ distance) of <4 Å and a Bürgi–Dunitz^32^ angle (*α*_BD_) of 76–115°. Monomers whose free energy minima lie outside a region in which the N_α_–C_*sp*2_ distance is less than 4 Å, even with an acceptable Bürgi–Dunitz angle, do not react efficiently. Metadynamics provided both high accuracy and full conformational sampling of monomers, as well as of the ribosome catalytic centre in the explicit solvent and ion environment. Metadynamics is fast, accurate and relatively cost-efficient. More importantly, it addresses multiple challenges impeding the ribosome-promoted biosynthesis of diverse heterooligomers. For applications in vivo, metadynamics can prioritize monomers for which orthogonal aminoacyl-tRNA synthetase variants are needed. For applications in vitro, it can identify monomers that are more likely to react within the PTC of wild-type ribosomes. In all cases, it can identify those monomers for which engineered ribosomes are needed. The work reported here should accelerate the in vivo ribosomal synthesis of much sought but not yet achieved sequence-defined, non-peptide heterooligomers and improve the diversity of the in vitro mRNA display campaigns used in drug discovery.

## Results and discussion

### An improved *E. coli* PTC structure informs reactivity

**Extended Data Fig. 2:**
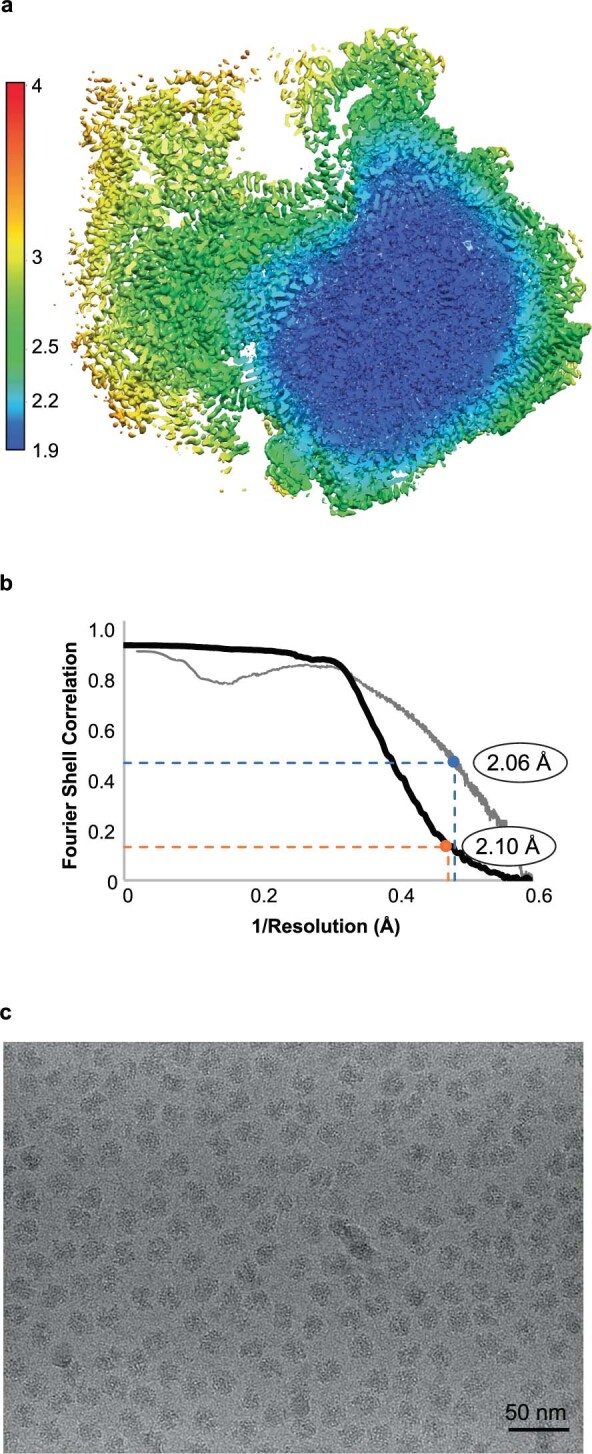
Resolution of the 50S subunit in the Met-Met ribosome structure. **a**, Local resolution of the 50S-focused cryo-EM map; **b**, FSC curves indicating resolution of the 50S subunit. Half-map FSC (black) is at 2.10 Å at the gold-standard cutoff value 0.143; map-vs.-model FSC (gray) is at 2.06 Å at the 0.5 cutoff. Only the large subunit was masked for the half-map FSC, and **c**, representative micrograph showing individual ribosome particles. The micrograph was low-pass filtered to 20 Å for easier visualization of particles.

We first established an improved PTC model that retained the high resolution of PDB 7K00 (ref. ^30^) and 6XZ7 (ref. ^31^) but included better-resolved α-amino acid monomers in the A and P sites (Fig. 1b). We reasoned that the low monomer density seen in 7K00 was due at least in part to aminoacyl-tRNA hydrolysis during grid preparation. To eliminate hydrolysis, we acylated 3′-amino tRNA^fMet^ with Met and *E. coli* methionyl-tRNA synthetase (MetRS)^33^. With this hydrolysis-resistant substrate, we obtained a 2.1-Å-resolution cryo-EM structure of the *E. coli* 50S subunit from 70S complexes, which contains well-resolved Met-NH-tRNA^fMet^ in both the A and P sites (Extended Data Fig. 2). During data processing, a classification approach more involved than our approach for PDB 7K00 (ref. ^30^) was used to determine a balance between maximal particle inclusion for higher global resolution and more discriminating exclusion of particles without well-resolved tRNA CCA ends (Methods and Supplementary Fig. 1).

**Extended Data Fig. 3:**
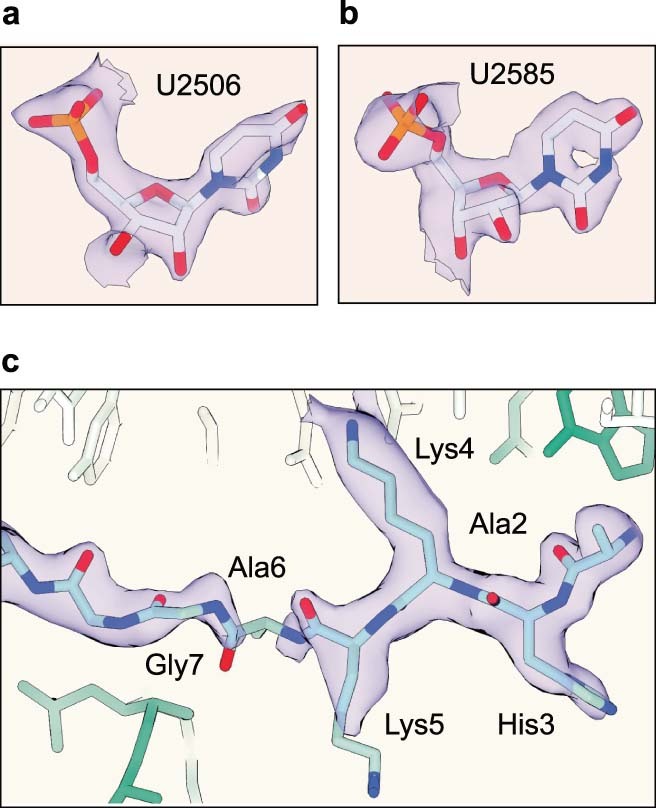
Structural model of U2506, U2585, and the N-terminus of ribosomal protein bL27. **a**, **b**, bases U2506 and U2585 of the 23S rRNA modelled in the cryo-EM density. The map here has been post-processed with a B-factor of −13 Å^2^ and supersampled for smoothness. **c**, Newly modelled section of r-protein bL27 (Ala-2 to Gly-7) (light blue). 23S rRNA is shown in white and A-site tRNA in green, with EM density shown in indigo. The map here has been post-processed with a B-factor of 10 Å^2^.

Local features of the PTC in the current map (Fig. 1b) are substantially improved from PDB 7K00 despite the lower 2.1 Å global resolution of the large subunit. This resolution was sufficient to improve the modelling of ordered water molecules, ions and polyamines in the PTC. Density for key bases U2506 and U2585 were modelled suboptimally in 7K00 due to poor density (Extended Data Fig. 1a), while in the current structure they have improved density best modelled in the favoured *anti* conformation in agreement with PDB 6XZ7 and 1VY4 (Extended Data Fig. 3a,b). The new map also enabled the modelling of residues 2–7 of bL27, the only ribosomal protein that is proximal to the tRNA CCA ends (Extended Data Fig. 3c).

**Extended Data Fig. 4:**
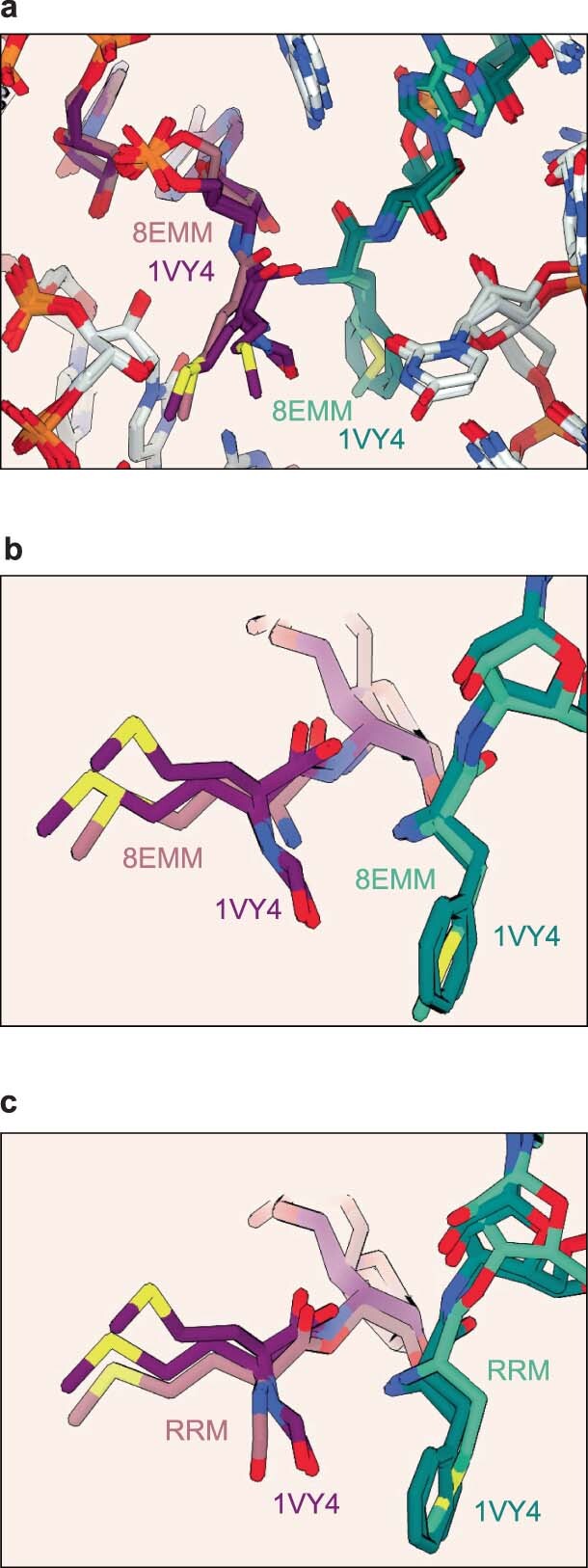
Orientation of the P-site monomer in the PTC is affected minimally by the presence or absence of a N-terminal formyl group. **a**, Superposition and **b**, close-up view of the PTC regions of 1VY4 (purple and teal) with that of 8EMM, the cryo-EM model reported here (rose and green). 23S rRNA is shown in white. Two structures are shown for 1VY4 because there are two ribosome structures in the unit cell. **c**, Superposition of the PTC regions of 1VY4 with the lowest energy pose of the metadynamics simulation of the RRM (in which the P-site tRNA has been altered in silico to carry fMet). The identity of the models 8EMM, 1VY4, and the RRM are indicated for both A and P sites with model−matched colors. These images reveal excellent superposition of the carbonyl groups (red) that are attacked during amide bond formation and the three P-site N atoms (in blue), regardless of whether that N is attached to protons alone or a formyl group. This analysis suggests that the presence or absence of a formyl group on the P-site tRNA has little or no effect on monomer placement within the PTC from the standpoint of structural biology.

Importantly for the simulations to follow, residues C75 and A76 in each acyl-tRNA are well resolved, with clear positioning of both the ribose and phosphate backbone as well as the amide linkages between Met and each A76 ribose (Fig. 1b). Beyond the amide linkage, the entirety of the A-site Met can be modelled. By contrast, the side chain and amine group of the P-site Met are not well resolved. However, the phosphate–ribose backbone of A76 in the P site is more clearly resolved than in previous models, resulting in a relative change in position (Extended Data Fig. 1c). In the model, the distance from the nucleophilic A-site amine N_α_ to the P-site carbonyl carbon C_*sp*2_ is 3.3 Å (Fig. 1b,c). The ability to visualize these elements is especially important for simulations, in which some remaining details of the residues themselves would be substituted in silico. Additionally, the placement of these atoms is in good agreement with previously reported PDB 1VY4 (Extended Data Fig. 4), which contains fMet in the P site. Although formylation of the initial Met residue is thought to facilitate correct positioning of fMet via hydrogen bonding with G2061 of the 23S rRNA, and a similar hydrogen-bonding interaction is seen clearly in a recent nascent chain-containing structure^34^, relatively poor density of the P-site monomer for both our map and 1VY4 suggests that a single Met residue in the P site remains disordered to some degree in the presence or absence of formylation.

### Establishing a test system for simulation studies

**Extended Data Fig. 5:**
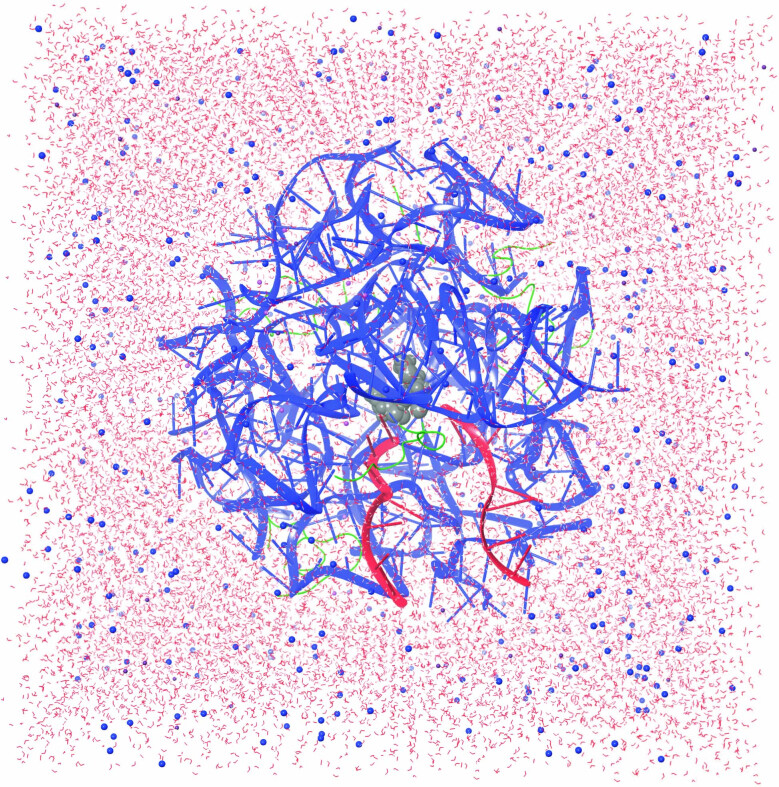
Image showing the simulation box used for molecular dynamics (MD) and metadynamics simulations. The 30 Å Reduced Ribosomal Model (RRM) is shown in the center in cartoon representation, wherein the RNAs of the 50S subunit are in blue, the protein residues are in green, the tRNAs are in red, and the monomers are in gray CPK representation. The water molecules are in red line representation, whereas the ions (K^+^, Cl^-^, Mg^2+^) are in blue spheres.

It is a well-established approximation, when judiciously applied, that the truncation and/or constraining of distant atoms of the ribosome can reduce computational cost while still providing insight about conformational dynamics within the PTC^35–37^. We thus truncated the cryo-EM model reported here to include only those residues within 30 Å of both the A-site and P-site Met. This truncated model was solvated in a water box and a physiological concentration of KCl was added. As the simulation environment was set up with preliminary coordinates from the cryo-EM structure, which did not yet contain ions or spermidine ligands, ordered Mg^2+^ ions from PDB 7K00 were rigid-body docked into the refined PTC coordinates. The final system contained ~88,000 atoms, including solvent, salt and counterions. This model is referred to as RRM, for the reduced ribosome model (Extended Data Fig. 5).

**Fig. 2:**
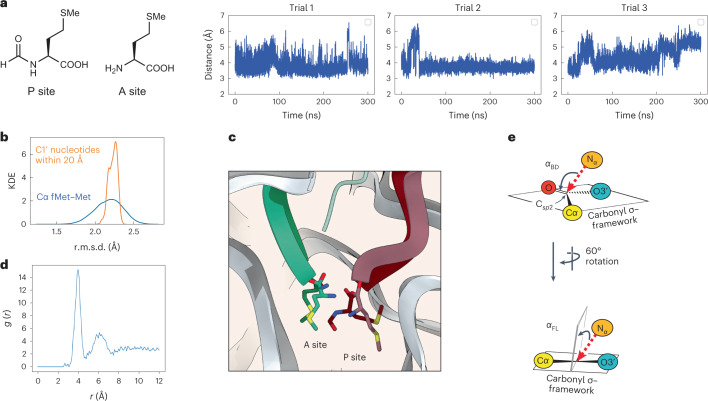
MD simulations of the input fMet–Met ribosome structural model. **a**, Trajectories illustrating the evolution of the distance between the A-site Met nucleophile (N_α_) and the P-site fMet carbonyl electrophile (C_*sp*2_) over 300 ns within the 30 Å RRM. **b**, Kernel density estimation (KDE) of the r.m.s.d. of either the C1′ nucleotides within the internal (non-fixed) 20 Å of the RRM (orange) or the Cα carbons of Met and fMet (blue) over the course of the simulation. **c**, Close-up of the bond-forming region of the PTC in the 2.1 Å cryo-EM model reported here (lighter shades) and a representative pose (Methods) of the simulation (darker shades) illustrating the relative positions of the Met and fMet monomers in the A and P sites, respectively. **d**, Radial distribution function *g*(*r*) indicates that the first and second solvation shells for the K^+^ and Mg^2+^ ions are within a distance (*r*) of ~4 and 6 Å of the P atoms of residues C75 and A76, respectively. **e**, Illustration of the Bürgi–Dunitz angle (*α*_BD_), which specifies the angle between the entering nucleophile and the C_*sp*2_=O double bond, and the Flippin–Lodge angle (*α*_FL_), which specifies the offset of the attack angle from the plane orthogonal to that defined by the carbonyl and the two adjacent substituents. σ = sigma. Source data

First, we set out to ensure that the RRM was stable during MD simulations and would recapitulate the conformations of the A-site and P-site monomers visualized by cryo-EM. We acylated the P-site Met with an *N*-formyl group in silico to serve as a more physiological initiator monomer, and positional restraints were placed on the Cα of every ribosomal protein residue and the C1′ of every nucleotide within the outer 10 Å of the 30 Å model. Test MD simulations (300 ns each) were run in triplicate, each with a different starting velocity, to evaluate statistical uncertainties and improve sampling (Fig. 2a). As an initial validation step, we examined backbone atom fluctuations during the three 300 ns simulations with respect to the original cryo-EM structure. We evaluated both overall model stability (the root-mean-square deviation (r.m.s.d.) of every unrestrained C1′ atom from its position in the cryo-EM structure) and monomer stability within the PTC (the r.m.s.d. of the Cα atoms of Met and fMet; Fig. 2b). Over the course of the simulation, the overall model r.m.s.d. was centred at 2.2 Å with a narrow distribution; stability within the PTC also averaged 2.2 Å, but with a slightly wider distribution. These metrics provide confidence that the overall RRM conformation and Met/fMet fluctuations remain stable (Fig. 2c).

As a further test of the validity of this model system, we examined the distribution of cations in the vicinity of the P-site tRNA 3′-CCA end. In the high-resolution structure determined here—after modelling of ions in the PTC was completed—and in the *Thermus thermophilus* structure (PDB 1VY4)^24^, a cation coordinates the phosphate group linking C75 and A76 of the P-site tRNA (Fig. 1b). Since the tRNA CCA ends were not well positioned in 7K00 (ref. ^30^), this Mg^2+^ ion was not included in the initial RRM. Notably, in test MD simulations that included K^+^ and Mg^2+^ ions, strong cation positioning close to the P-site tRNA C75 and A76 phosphates is observed. The radial distribution functions for K^+^ and Mg^2+^ ions show ion to phosphorus distances consistent with phosphate coordination during the time course (Fig. 2d).

We next examined the geometric relationship between the A-site and P-site monomers that react to promote peptide bond formation. This geometric relationship is described by three metrics. The first is the distance between the candidate nucleophilic α-amine of the A-site monomer (N_α_) and the candidate electrophile, the ester carbonyl carbon of the P-site monomer (C_*sp*2_). This N_α_–C_*sp*2_ distance is 3.3 Å in the 2.1-Å-resolution cryo-EM structure reported here; it averaged 4.0 ± 0.3 Å throughout the three simulations. The second and third metrics are angles that define the approach of the nucleophile relative to the carbonyl (Fig. 2e). The Bürgi–Dunitz angle (*α*_BD_)^32^ specifies the angle between the entering nucleophilic N_α_ and the C_*sp*2_=O double bond. Reported values^32,38^ for *α*_BD_ vary from ~105° to ~90°. The Flippin–Lodge angle (*α*_FL_)^39,40^ specifies the offset of the attack angle from the plane orthogonal to that defined by the carbonyl and the two adjacent substituents and is smaller, often <20°. The values of *α*_BD_ and *α*_FL_ derived from the cryo-EM structure reported here are 98° and 19°, respectively, also in line with canonical data and predictions. The average values of *α*_BD_ and *α*_FL_ derived from the simulations are centred at 94° ± 15° and 38° ± 12°, respectively.

### Evaluation of non-l-α-amino acid monomers

**Fig. 3:**
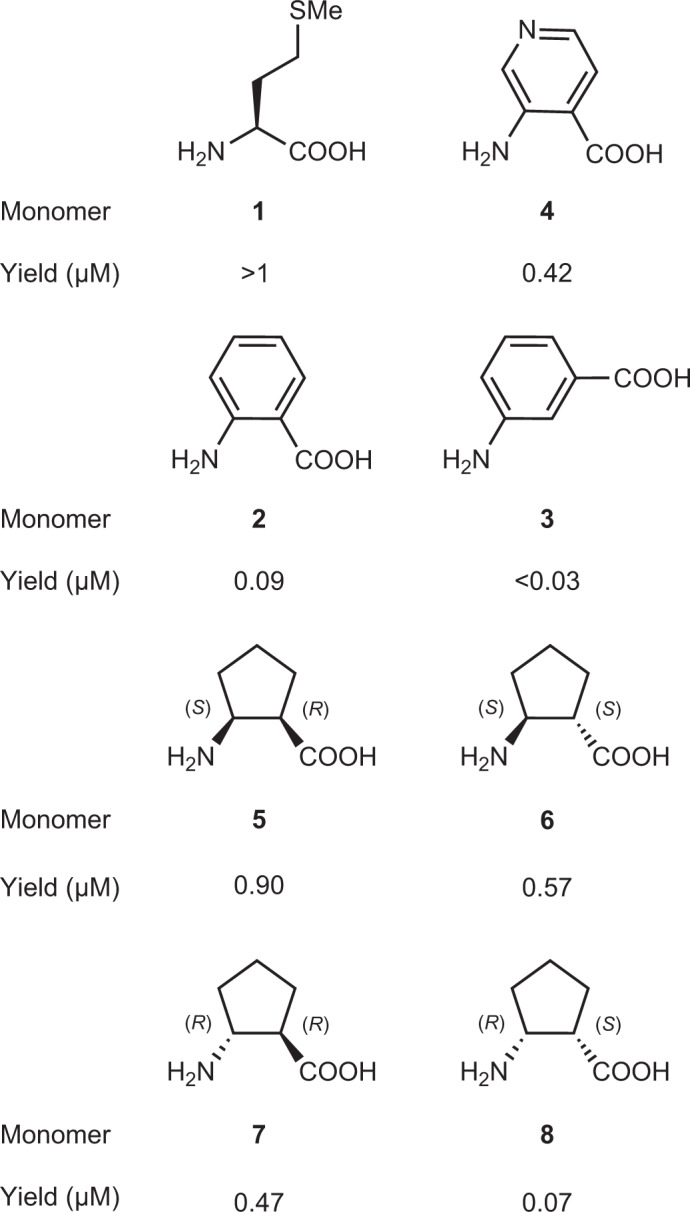
Chemical structures of monomers tested in MD and metadynamics simulations. Structures of aminobenzoic acid and cyclic β^2,3^-2-aminocyclopentane-1-carboxylic acid monomers and data from in vitro translation assays are shown. The yield shown represents the relative efficiency of translation of DNA template fMet-Trp-Lys-Lys-Trp-Lys-Lys-Trp-Lys-X-Gly-Asp-Tyr-Lys-Asp-Asp-Asp-Asp-Lys (for aminobenzoic acids) or fMet-Trp-Lys-Lys-Trp-Lys-Lys-Trp-Lys-Phe-X-Gly-Asp-Tyr-Lys-Asp-Asp-Asp-Asp-Lys (for cyclic β^2,3^-2-aminocyclopentane-1-carboxylic acid monomers). In vitro translation reaction conditions were comparable within each monomer class, as was the efficiency of tRNA acylation. Because the templates differ, comparisons are limited to those within a given monomer class. The value for Met was estimated at 2.5 times the efficiency of Ala.

With a validated workflow in hand, we evaluated the intra-PTC conformational landscapes of two sets of non-l-α-amino acid monomers (Fig. 3). These monomers exhibit differences in overall structure, stereochemistry and basicity of the nucleophilic atom. All the monomers acylate a common tRNA with a high yield in flexizyme (ref. ^41^)-promoted reactions, yet within a given set, the acylated tRNAs that result differ substantially in translational efficiency in vitro using *E. coli* ribosomes. The first monomer set comprises a trio of aminobenzoic acid derivatives (**2**–**4**) that introduce extended, *sp*^2^-hybridized aromatic backbones into translated polypeptides. Of these three monomers, *ortho*-substituted pyridine **2** is the most reactive, with a translation yield at least fourfold higher than reactions of tRNA acylated with *ortho*-aminobenzoic acid **3** (ref. ^13^). The translation yield using *meta*-aminobenzoic acid **4** was almost undetectable. The second monomer set comprises all four stereoisomers of the cyclic β^2,3^-amino acid 2-aminocyclopentane-1-carboxylic acid (**5**–**8**) (ref. ^10^), studied extensively as foldamers^42^. Of these four monomers, stereoisomer **5** is the most reactive; the translation yield was more than tenfold higher than that of enantiomer **8**. The translational efficiencies of the enantiomeric pair **6** and **7** were moderate.

### N_α_–C_*sp*2_ distance is not a predictor of reactivity

**Fig. 4:**
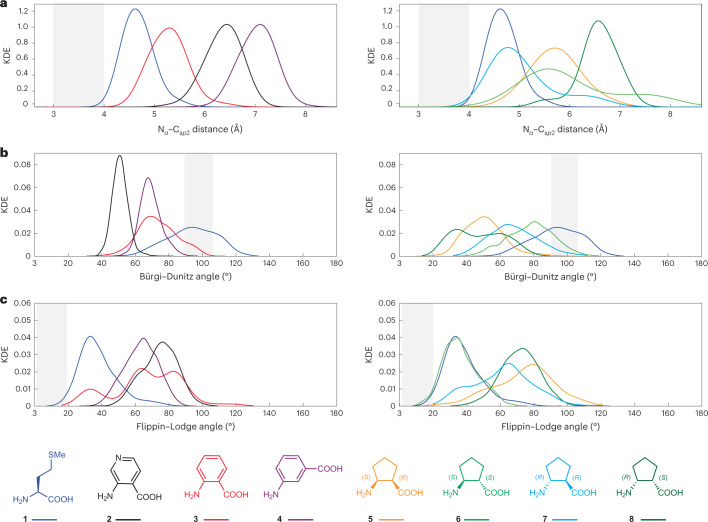
Kernel density estimation of the three geometric measurements from MD simulations. **a**–**c**, N_α_–C_*sp*2_ distance (**a**), Bürgi–Dunitz angle (**b**) and Flippin–Lodge angle (**c**) for aminobenzoic acids (left) and β^2,3^-amino acid 2-aminocyclopentane-1-carboxylic acid monomers (right) within the RRM over the course of the 300 ns simulation. The grey zones highlight N_α_–C_*sp*2_ distances of between 3 and 4 Å, and values of *α*_BD_ and *α*_FL_ between 90° to 105° and 5° to 20°, respectively. Source data

**Extended Data Fig. 6:**
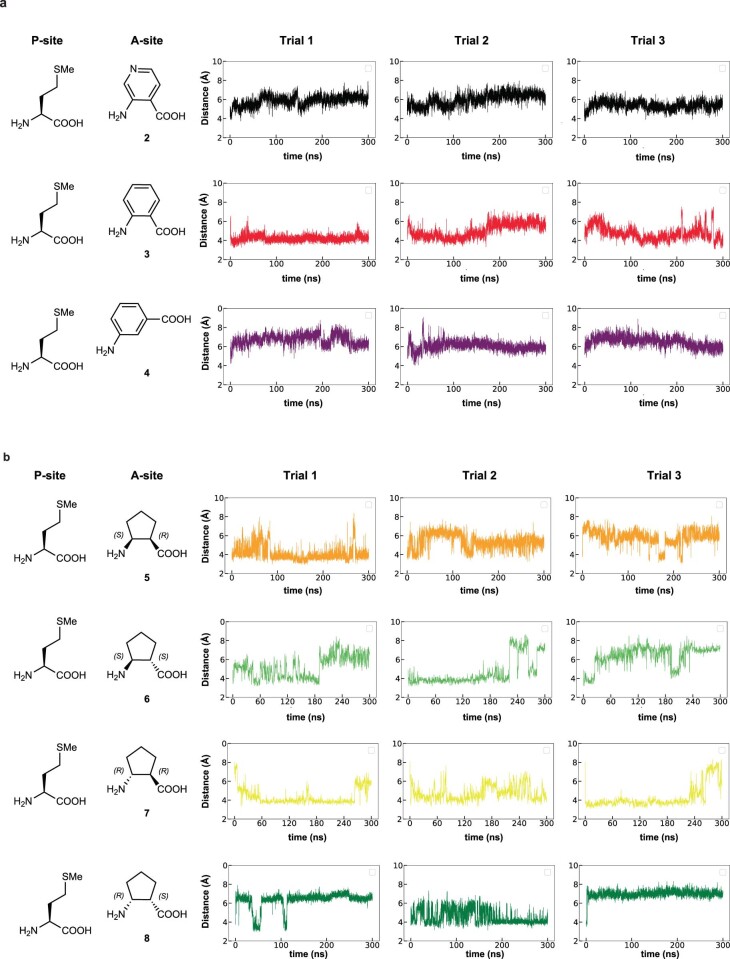
Trajectories from MD simulations of different monomers in the A site. Trajectories illustrating evolution of the distance between the A-site nucleophile (Nα) and the P-site fMet carbonyl electrophile (Csp2) over 300 ns within the 30 Å Reduced Ribosome Model (RRM). **a**, aminobenzoic acids **2–4**; **b**, β^2,3^-2-aminocyclopentane−1-carboxylic acids **5–8**. We used Schrödinger’s Force Field Builder (Schrödinger release 2022-1) to parametrize the existing OPLS4 force field to accurately describe the conformational dynamics of each new monomer. Maestro’s 3D Builder was then used to assemble the new ester bond and minimize the system to release clashes with surrounding residues. Source data

**Extended Data Fig. 7:**
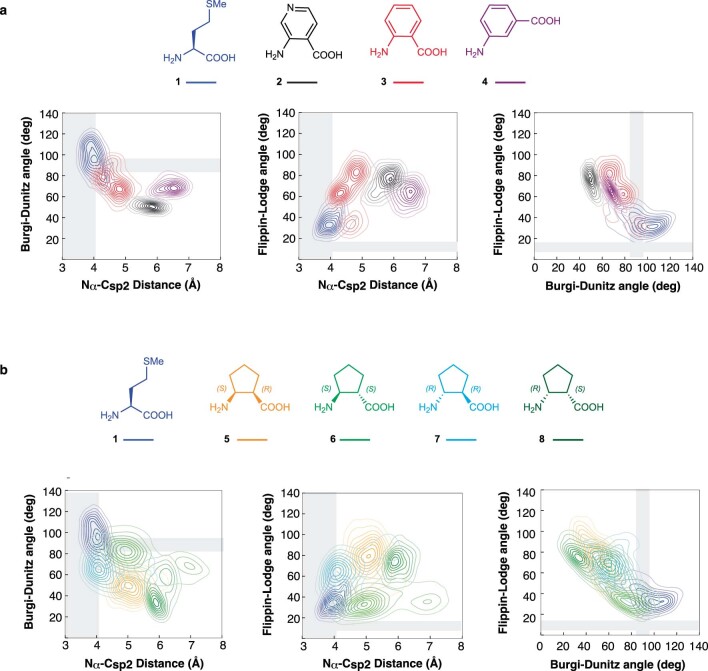
Scatter plots of the three geometric measurements used to evaluate conformational fluctuations. **a**, fluctuations of aminobenzoic acids **2–4** and **b**, fluctuations of cyclic β^2,3^-amino acid 2-aminocyclopentane-1-carboxylic acid monomers **5–8** in comparison to Met **1** in the PTC of the RRM throughout the MD runs are shown. Specifically, the Nα–Csp2 distance is plotted against either the Bürgi–Dunitz angle αBD (left) or the Flippin-Lodge angle αFL (center), and the two angles are plotted against each other (right). The gray zones highlight Nα – Csp2 distances of between 3 and 4 Å, and values of αBD and αFL of between 90° and 105° and 5° to 20°, respectively. Source data

Next, we evaluated in triplicate the evolution of each non-l-α-amino acid monomer over 300 ns within the PTC of the 30 Å RRM. We evaluated changes in the distance between N_α_ and C_*sp*2_ as well as the values of *α*_BD_ and *α*_FL_ (Fig. 4 and Extended Data Fig. 6). A plot of the average kernel density estimate as a function of all three metrics spans a wide range for both monomer sets (Fig. 4a). As described above, simulation of the native fMet–Met pair reproduces the expected values for the N_α_–C_*sp*2_ distance (4.0 ± 0.3 Å) and reasonable values of both *α*_BD_ (94° ± 15°) and *α*_FL_ (38° ± 12°). However, the midpoint N_α_–C_*sp*2_ distance for non-l-α-amino acid monomers **2**–**8** does not correlate with reactivity. For aminobenzoic acids **2**–**4**, although the midpoint N_α_–C_*sp*2_ distance for *ortho*-aminobenzoic acid **3** (4.7 Å) is smaller than that of *meta*-aminobenzoic acid **4** (6.4 Å), it is also smaller than that of the pyridine analogue **2** (5.7 Å), which is by far the most reactive in this monomer set. In the case of cyclic β^2,3^-amino acids, reactivity again fails to track with midpoint N_α_–C_*sp*2_ distance. Reactivity tracks in the order **5** > **6** ≈ **7** ≫ **8**, whereas the midpoint N_α_–C_*sp*2_ distance tracks in the order **7** < **5** ≈ **6** < **8**. Similar conclusions can be drawn when *α*_BD_ and *α*_FL_ are considered in place of the midpoint N_α_–C_*sp*2_ distance (Fig. 4b,c and Extended Data Fig. 7).

### Metadynamics widely samples intra-PTC monomer conformation

Based on the lack of correlation discussed above, we concluded that in the absence of experimentally determined starting structures for non-l-α-amino acid monomers, the in silico assembled starting conformations could be trapped in local energy wells and likely did not explore the entirety of conformational space during the course of the MD simulation. This problem is common in MD simulations of biomolecular systems—sometimes the sampling over a given time scale is insufficient, whereas at other times the system may be trapped at a local energy minimum and diffuse slowly, and at still other times both of these scenarios occur simultaneously^27,43,44^. To achieve a more thorough sampling of the monomer-dependent conformational landscape within the PTC, we turned to metadynamics^45^, which has been used extensively in the sampling of biomolecular simulations^46^. Metadynamics improves sampling by introducing an additional force (bias potential) on a chosen number of and chosen types of degrees of freedom known as collective variables. For practical purposes, it is important to limit the number of collective variables, while choosing collective variables that efficiently sample the desirable conformational space^47,48^. We thus chose two collective variables: the N_α_–C_*sp*2_ distance and the *α*_BD_ value. The *α*_BD_ value was chosen in preference to *α*_FL_ because it varies over a wider range among the monomers evaluated (Extended Data Fig. 7).

### Metadynamics recapitulates monomer relative reactivity

**Fig. 5:**
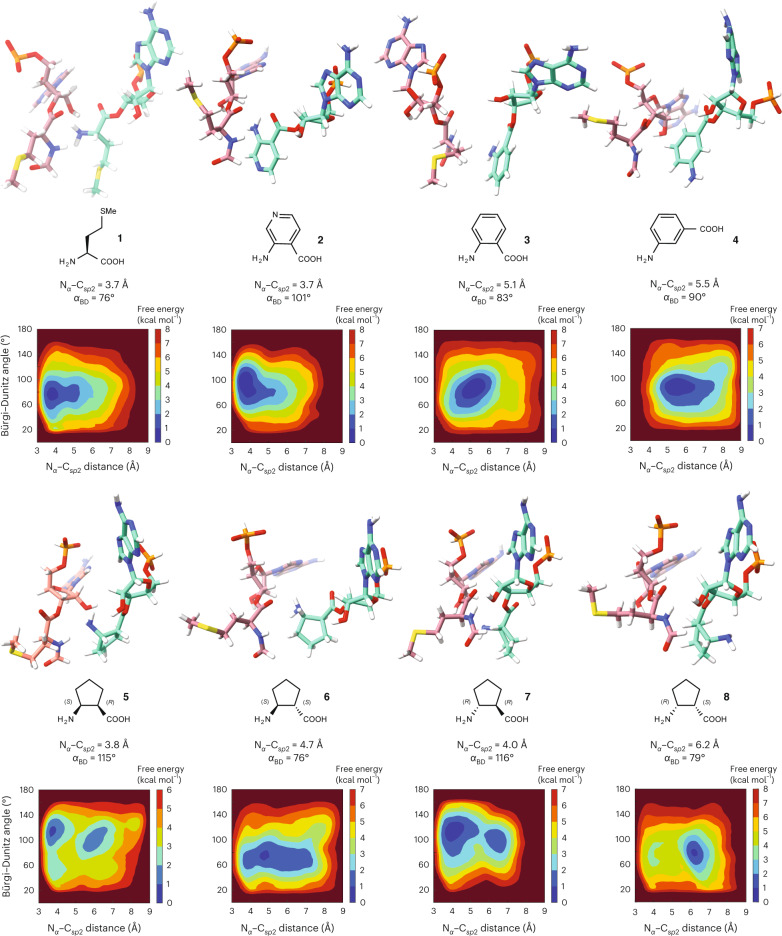
Plots of FESs from metadynamics simulations. FESs of 30 Å RRM containing a P-site tRNA acylated with fMet and A-site tRNAs acylated with monomers **1**–**8**, plotted along the collective variables of Bürgi–Dunitz angle *α*_BD_ and N_α_–C_*sp*2_ distance. The metadynamics simulations that generated each FES began with the 2.1 Å cryo-EM model reported here, but the A-site Met was in silico mutated to the other monomers, while the P-site Met was converted to fMet. Each FES shown is the average of two metadynamics runs starting from orientations of the A-site monomers that differ by a 180° rotation about the ψ angle. The colour scale represents the free energy in kilocalories per mole, where the global minima are set at 0 and therefore the various heights of the energy scales are based on the energetics of the fluctuations of the A-site monomers. The conformation and relative geometry of the P-site (rose) and A-site (green) monomers at the free energy minimum are shown above each plot. Poses were chosen to highlight the N_α_–C_*sp*2_ distance and the Bürgi–Dunitz angle *α*_BD_ at the free energy minimum. The tRNAs to which each monomer is attached as well as the RRM surrounding the two monomers have been omitted for clarity. Coordinates for the lowest energy models obtained for each simulation are available at 10.5281/zenodo.7730661. Two methods were used to establish that convergence was reached during the 100 ns runs. First, trajectory plots representing the N_α_–C_*sp*2_ distance and Bürgi–Dunitz angle (*α*_BD_) for metadynamics replicas reveal that the conformation space is revisited multiple times over the course of the metadynamics runs (Supplementary Figs. 2 and 3). Second, FESs calculated for the last 80 ns of the 100 ns simulation are qualitatively similar to those calculated after 100 ns, with the differences between the FESs of the last 80 ns and after 100 ns on average ~0.8 kcal mol^–1^ (Supplementary Figs. 4–6). Source data

We began the metadynamics simulations with two initial conformations of each monomer. One conformation was identical to that used to initiate unbiased MD simulations (Fig. 4). The second conformation was altered by rotating the psi (ψ) angle (angle between the Cα and the C_*sp2*_ of the monomers) by 180°, to generate an alternative initial position of the nucleophile amine N_α_ relative to the C_*sp*2_ of fMet. Examination of plots showing *α*_BD_ as a function of the N_α_–C_*sp*2_ distance after the metadynamics simulations reveals minima that clearly differentiate highly reactive and less reactive monomers within each monomer set (Fig. 5). The metadynamics free energy surface (FES) contour plot of a RRM containing P-site fMet and A-site Met monomers shows excellent agreement with the N_α_–C_*sp*2_ distance and *α*_BD_ values determined by cryo-EM. The global minimum N_α_–C_*sp*2_ distance is centred at 3.7 Å, and the global minimum value of *α*_BD_ is centred at 76°, while the fluctuations within 1 kcal mol^–1^ reach values between 3.4–4.4 Å and 61–90°, respectively. These values show good agreement with both unbiased MD results (4 Å and 94°) and the metrics derived from the high-resolution structure reported here (3.3 Å and 93°).

More importantly, the global minima for the monomer within each set that reacts most efficiently within the PTC, notably pyridine **2** and (1*R*,2*S*)-2-aminocyclopentane carboxylic acid **5**, populate conformations within the PTC with N_α_–C_*sp*2_ distances centred at or below 3.8 Å and *α*_BD_ values between 101° and 115°. By contrast, monomers that are relatively inactive, notably *ortho*- and *meta-*aminobenzoic acids **3** and **4** and (1*S*,2*R*)-2-aminocyclopentane carboxylic acid **8** (the enantiomer of **5**), populate conformations with N_α_–C_*sp*2_ distances between 5.1 and 6.2 Å, with *α*_BD_ values centred at 84°. The two moderately active 2-aminocyclopentane carboxylic acid isomers **6** and **7** populate a conformational space defined by either long N_α_–C_*sp*2_ distances (**6**) or dual conformational minima (**7**).

**Extended Data Fig. 8:**
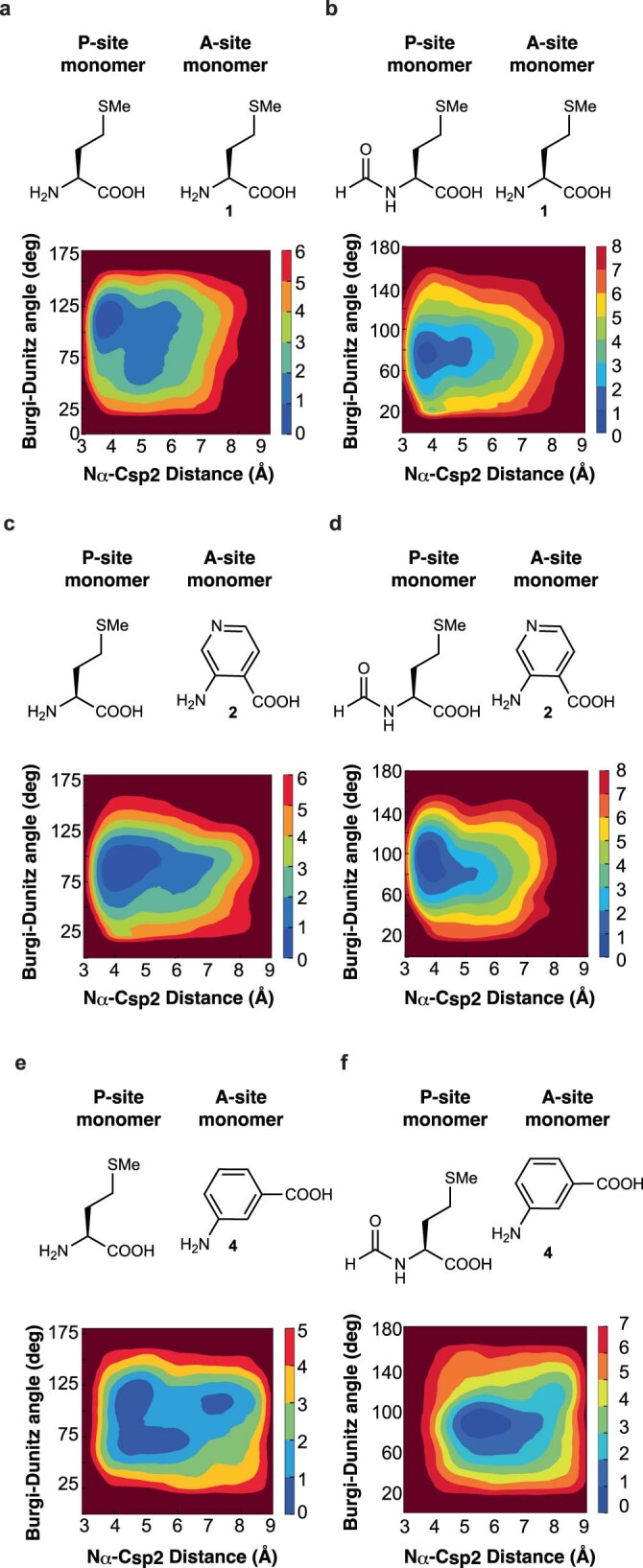
Plots of free energy surfaces from metadynamics simulations: the effect of a P-site N-terminal formyl group. Free energy surfaces (FES) (kcal/mol) of the 30 Å RRM model containing an P-site tRNA acylated with **a, c, e**, Met or **b, d, f**, fMet plotted along the Bürgi–Dunitz angle αBD and Nα–Csp2 distance collective variables. A-site tRNAs were acylated with **a, b** Met **1**; **c, d**, aminobenzoic acid **2**; **e, f**, aminobenzoic acid **4**. Source data

We also carried out additional metadynamics simulations of the RRM in which the P-site monomer was Met (not fMet) and the A-site monomer was either aminobenzoic acid **2** (highly reactive) or aminobenzoic acid **4** (poorly reactive; Extended Data Fig. 8c–f). In this case, when the simulations begin without an *N*-formyl group, monomer **4** shows a more significant change in its FES than does monomer **2**. As a result, monomer **4** shows a more favourable energy minimum (in terms of distance) for the reaction to occur, and the differences between the minimum energy poses are not as large as when the simulation begins with an N-terminal fMet. This result provides additional support for our decision to formylate the P-site Met in silico prior to initiating simulation. Regardless, these data suggest that the most reactive aramid monomers significantly populate a conformational space characterized by an average N_α_–C_*sp*2_ distance of <4 Å and an average *α*_BD_ of between 76° and 115°.

**Fig. 6:**
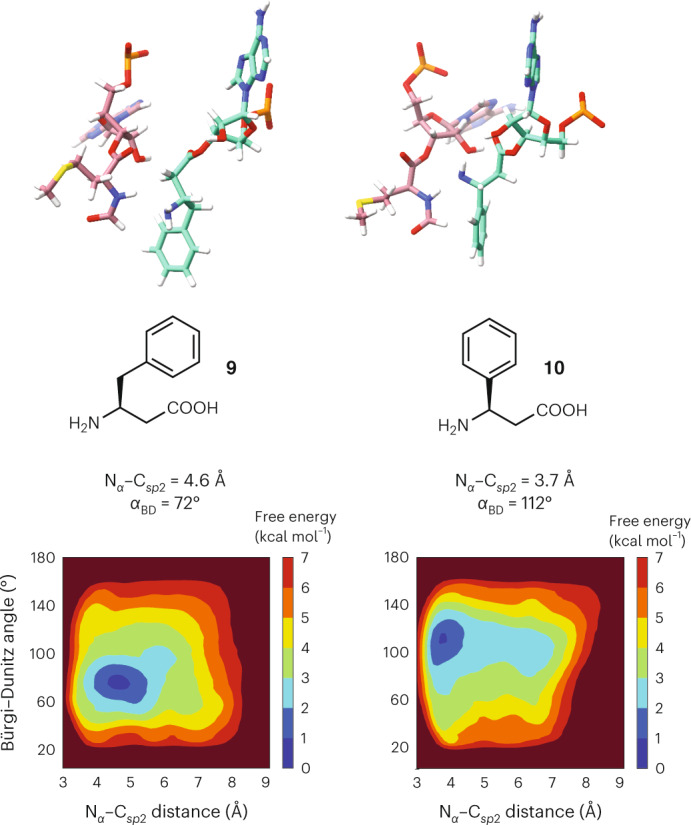
Metadynamics analysis of structurally unrelated monomers. The workflow accurately predicts the relative reactivities of two monomers that are structurally unrelated to monomers **2**–**8**, notably the less reactive (*S*)-β^3^-homophenylalanine **9** and the more reactive (*S*)-β^3^-homophenylglycine **10**. Shown are the FESs produced by the metadynamics workflow as well as the poses and geometries of the two systems at the global minimum. Source data

Encouraged by these results, we next asked whether we could apply the metadynamics workflow to classify the relative reactivities of two structurally unrelated monomers that possess greater conformational freedom than those evaluated initially—notably (*S*)-β^3^-homophenylalanine **9** and (*S*)-β^3^-homophenylglycine **10**. These monomers acylate an identical tRNA with identical efficiencies in flexizyme-promoted reactions, but (*S*)-β^3^-homophenylglycine **10** is noticeably more reactive in in vitro translation reactions^49^. Application of the metadynamics workflow outlined above for monomers **2**–**8** to monomers **9** and **10** generated a pair of FESs that clearly differentiated the two monomers in a manner consistent with their reactivity (Fig. 6). The global minimum of the FES generated for a RRM containing monomer **10** in the A site mirrors that of reactive monomers **2** and **5**, notably a N_α_–C_*sp*2_ distance centred at or below 4 Å (3.7 Å) and *α*_BD_ of 111°. By contrast, the global minimum for monomer **9** mirrors that for inactive monomer **4**, notably a N_α_–C_*sp*2_ distance greater than 4.6 Å and a low *α*_BD_ of 72°.

## Conclusions

In summary, here we implement a structure-informed, physics-based workflow to reliably classify the reactivity of non-l-α-amino acid monomers in amide-bond-forming reactions catalysed by the *E. coli* ribosome. Non-l-α-amino acid monomers that populate a conformational space characterized by N_α_–C_*sp*2_ distances of ≤4 Å and Bürgi–Dunitz angles of 76° and 115° react efficiently within the PTC of wild-type *E. coli* ribosomes. These predictions hold for three diverse families of molecules: aminobenzoic acids, β^3^-amino acids and β^2,3^-cyclic amino acids. Monomers that cannot appreciably occupy a region in which the N_α_–C_*sp*2_ distance is less than 4 Å, even with an acceptable Bürgi–Dunitz angle, do not react. The experimental data, along with the models that emerge from the simulations, are both consonant with notions about ‘near-attack/near in-line attack’ transition states in biocatalysis^50^. Importantly, these bond-forming trajectories intersect with the stereoelectronic considerations associated with our analysis of Bürgi–Dunitz (*α*_BD_) and Flippin–Lodge (*α*_FL_) angles. Accordingly, even in the absence of a high-level quantum mechanical treatment of these in-ribosome transition states, the convergence of the experimental data and simulations with the observed incorporation efficiencies of natural and non-natural non-l-α-amino monomers provides a self-consistent picture of accessible geometries for efficient incorporation. More broadly, the metadynamics workflow implemented herein addresses two barriers impeding the ribosome-promoted biosynthesis of diverse heterooligomers. For applications in vivo, metadynamics can prioritize monomers for which orthogonal aminoacyl-tRNA synthetase variants are needed. For applications in vitro, metadynamics can identify monomers that are likely to react within the PTC of wild-type ribosomes, and those for which engineered ribosomes are needed.

## Methods

### Preparation of acylated tRNA

#### Synthesis of N-1 tRNA^fMet^

tRNAs missing the 3’ terminal nucleotide are referred to here as N-1 ‘tRNAs’. Templates for in vitro transcription were prepared using a double polymerase chain reaction (PCR) amplification method. Base C1 was mutated to G for optimal T7 RNA polymerase initiation. First, long overlapping primers tRNA-fMet-C1G_temp F and RNA-fMet-C1G-A_temp R (Supplementary Table 1) were PCR amplified using Q5 DNA polymerase from New England Biolabs (NEB). Products were gel purified and amplified using short primers tRNA-fMet-C1G_amp F and tRNA-fMet-C1G-A_amp R (Supplementary Table 1). The second base of each reverse primer in this step was modified with 2′OMe to prevent nonspecific addition by T7 RNA polymerase^51^. Amplification PCRs were ×1 phenol chloroform extracted, ×2 chloroform washed and precipitated with ×3 volumes of ethanol (EtOH). DNA quantity was measured on an agarose gel using a known standard.

T7 in vitro transcription was performed in a buffer containing 50 mM Tris-HCl, pH 7.5; 15 mM MgCl_2_; 5 mM dithiothreitol (DTT); and 2 mM spermidine. Reactions contained 2.5 mM each nucleoside triphosphate (NTP), 1:40 NEB murine RNase inhibitor, 25 μg T7 RNA polymerase (gift from B. Martinez, Cate Lab), 0.0005 U µl^–1^ pyrophosphatase (PPase) and ~1,000 ng DNA template per 100 μl of transcription reaction. Reactions were incubated for 16 h at 37 °C; treated with 1/20th volume RQ1 deoxyribonuclease (DNase; 1 U µl^–1^ stock) for 30 min at 37 °C; and precipitated with 1/10th volume of 3 M NaOAc, pH 5.2, and ×3 volumes of EtOH. Precipitated tRNA was pelleted, washed once with 70% EtOH and resuspended in loading dye (95% formamide, 40 mM EDTA, 0.05% bromophenol blue, 0.05% xylene cyanol).

Gel purification was performed using ~20-cm-long 12% polyacrylamide ×1 TBE 7 M urea gels poured roughly 2 mm thick. Bands were excised using UV shadowing, crushed, frozen on dry ice briefly (with elution buffer) and eluted at 4 °C overnight in 300 mM NaOAc, pH 5.2; 1 mM EDTA; and 0.5% (w/v) sodium dodecyl sulfate (SDS). Approximately 2 ml buffer was used per 500 μl transcription reaction. Eluted tRNA was pipetted off the gel debris and precipitated with 1 µl GlycoBlue Coprecipitant and ×3 volumes of EtOH. Pelleted tRNA was resuspended in water and stored at −80 °C.

#### Purification of *Archaeoglobus fulgidus* CCA-adding enzyme

The full CCA-adding enzyme gene was purchased from Twist Bioscience and cloned into a plasmid carrying a T7 promoter and C-terminal 6x histidine tag. The resulting plasmid was transformed into BL21 (DE3) Rosetta2 pLysS cells for expression. Some 2 × 1 l of lysogeny broth (LB) + 100 μg ml^–1^ ampicillin were induced with a 1:100 dilution of overnight culture (grown at 37 °C). Cells were grown at 37 °C until the optical density measured at a wavelength of 600 nm (OD_600_) was ~0.5, and induced with 0.5 mM isopropylthio-β-galactoside (IPTG) for 3 h. Cells were pelleted and washed with lysis buffer (20 mM HEPES buffer, pH 7.5; 150 mM NaCl; 10 mM MgCl_2_; 1 mM DTT; 20 mM imidazole) and stored at −80 °C. For lysis, cells were resuspended in ~30 ml lysis buffer with a tablet of Pierce EDTA-free protease inhibitor. Cells were lysed using a sonicator to deliver ~8,000 J of energy, and lysate was clarified at 18,000 r.p.m. in a JA-20 rotor (Beckman) for 30 min at 4 °C. Supernatant was applied to a 1 ml HisTrap column recirculating for ~30 min. The bound lysate was fractionated in a fast protein liquid chromatography system with five column volumes of lysis buffer and 10 column volumes of linear elution gradient from lysis buffer to lysis buffer, with 500 mM imidazole. Then 0.5 ml fractions were collected and analysed using SDS–polyacrylamide gel electrophoresis (SDS-PAGE). Fractions containing CCA-adding enzyme were combined and concentrated in a 30,000 Da molecular weight cut-off spin filter and buffer exchanged into buffer containing 20 mM HEPES, pH 7.5; 20 mM KCl; 1 mM EDTA; and 2 mM β-mercaptoethanol (BME). Protein was applied to a 1 ml heparin column and processed on a fast protein liquid chromatography system, as previously described^52^. Protein-containing fractions were combined and dialysed against 20 mM HEPES, 150 mM NaCl, 10 mM MgCl_2_, 1 mM DTT and 20% glycerol, and stored at −80 °C. The final concentration was 54 μM in ~1 ml (extinction coefficient estimated at 55,350 M^−1^ cm^−1^).

#### 3′NH_2_-ATP-tailing of N-1 tRNAs

The procedure used for tRNA amino-tailing was adapted from ref. ^53^. Equimolar amounts of N-1 tRNA and *A. fulgidus* CCA-adding enzyme (usually 2 μM) were combined in a reaction containing 100 mM glycine, pH 9; 10 mM MgCl_2_; 1 mM DTT; 0.002 U µl^–1^ PPase; and 0.5 mM 2′-NH_2_-ATP (2′-amino-2′-deoxyadenosine-5′-triphosphate, purchased from Axxora). Reactions were incubated at 37 °C for 2 h. Degradation of tRNAs was seen if reactions were incubated past completion. NH_2_-tRNAs were extracted with 1:10 volume of 3 M NaOAc, pH 5.2, and one volume of acidic phenol chloroform; cleaned twice with one volume of chloroform; and precipitated with three volumes of EtOH. NH_2_-tRNAs were resuspended in water, and the reaction yield was analysed on a 10% acrylamide 7 M TBE urea gel (20 cm). Some 1–2 pmol of tRNA was loaded per lane, and the gel was stained with SYBR Green II after running. NH_2_-tRNAs were stored in aliquots at −80 °C.

#### Purification of MetRS

The 6xHis-tagged MetRS expression plasmid was a gift from P. Ginther (Schepartz lab, University of California, Berkeley). The plasmid was transformed into BL21 (DE3) Codon+ RIL cells (T7 promoter). Culture overnights were diluted into ZYM-5052 autoinducing media^54^ and expressed overnight at 37 °C. Cells were pelleted and resuspended in lysis buffer (20 mM Tris, pH 7.8; 150 mM NaCl; 5 mM imidazole; 0.5 mM EDTA; ~35 ml). Cells were lysed with a sonicator on ice until ~8,000 J had been delivered to the sample. Lysate was clarified by centrifugation at 18,000 r.p.m. (JA-20 rotor; Beckman) at 4 °C for 30 min. Supernatant was applied to a 5 ml HisTrap column, and the column was attached to a fast protein liquid chromatography system. Protein was purified on the fast protein liquid chromatography system by washing with five column volumes of lysis buffer with 23 mM imidazole and eluting with a linear gradient of 20 column volumes from 23–500 mM imidazole. Fractions containing the desired protein were pooled; dialysed overnight against 50 mM HEPES, pH 7.5, plus 100 mM KCl, 10 mM MgCl_2_, 7 mM BME and 30% glycerol; and then concentrated in spin filters. Protein was stored at −80 °C in aliquots.

#### Aminoacylation of tRNAs

All aminoacylation reactions were performed in a buffer containing 50 mM HEPES, pH 7.5; 20 mM MgCl_2_; 10 mM KCl; 2 mM DTT; 10 mM ATP; 1:40 volume RNase inhibitor (murine, NEB); and 5–10 mM amino acid. Aminoacylation enzymes were usually used at 1 μM, and tRNAs, at 2–5 μM depending on stock concentration. Generally, a 1:5 enzyme/tRNA ratio was not exceeded. Reactions were incubated at 37 °C for 30 min and then extracted and precipitated as described in the previous section ‘3′NH_2_-ATP-tailing of N-1 tRNAs’.

### 70S ribosome preparation: Met–Met ribosome complex formation

All steps towards the Met–Met ribosome structure were performed essentially as described^30^. Briefly, the complex was formed by incubating 100 nM 70S ribosomes, 5 μM mRNA, ~1.6 μM Met-NH-tRNA^fMet^ and 100 μM paromomycin in buffer AC (20 mM Tris, pH 7.5; 100 mM NH_4_Cl; 15 MgCl_2_; 0.5 mM EDTA; 2 mM DTT; 2 mM spermidine; 0.05 mM spermine) for 30 min at 37 °C. The mRNA sequence GUAUAA**GGAGG**UAAAAAUGAUGUAACUA was synthesized by Integrated DNA Technologies (Shine–Dalgarno sequence in bold; ×2 AUG codons underlined).

### Cryo-EM sample preparation

The sample was prepared for imaging on 300 mesh R1.2/1.3 UltrAuFoil grids with an additional layer of float-transferred amorphous carbon support film. The grids were washed in chloroform prior to carbon floating. Before applying the sample, grids were glow discharged in a PELCO easiGlow at 0.39 mbar and 25 mA for 12 seconds. Some 4 μl of sample was deposited onto each grid and left for 1 minute. The grid was then washed with a buffer containing 20 mM Tris, pH 7.5; 20 mM NH_4_Cl; 15 MgCl_2_; 0.5 mM EDTA; 2 mM DTT; 2 mM spermidine; and 0.05 mM spermidine by successively touching it to three 100 μl drops of the buffer immediately prior to freezing. Grids were blotted and plunge-frozen in liquid ethane with an FEI Mark IV Vitrobot using the following settings: 4 °C, 100% humidity, blot force 6 and blot time 3. Grids were clipped for autoloading and stored in liquid N_2_.

### Cryo-EM data collection

Cryo-EM data collection parameters are summarized in Supplementary Table 2. Dose-fractionated videos were collected on a Titan Krios G3i microscope at an accelerating voltage of 300 kV and with a BioQuantum energy filter. Videos were recorded on a GATAN K3 direct electron detector operated in CDS mode. A total dose of 40 e^–^ Å^–2^ (e^–^, electron) was split over 40 frames per video. The magnification was 102,519 for a physical pixel size of 0.8296 Å and super-resolution pixel size of 0.4148 Å (based on a pixel size calibration performed after the final structure was obtained). Data collection was automated with SerialEM (ref. ^55^) v.3.8.2, which was also used for astigmatism correction by CTF and coma-free alignment by CTF. One video per hole was collected using stage shift to move between the centre holes of a 3 × 3 hole template and image shift to collect on the surrounding eight holes. The defocus ramp was set to range between −0.5 and −2 μm.

### Image processing

RELION steps were performed in RELION v.3.1 and the beta of v4.0 (refs. ^56,57^). Motion correction was performed with MotionCor2 (ref. ^58^) v.1.0.1 within the RELION graphical user interface, and micrographs were binned to the physical pixel size (believed to be 0.81 Å at the outset). Contrast transfer function (CTF) parameters were estimated using CTFFind4 (ref. ^59^) v.4.1.5. Micrographs with poorly fitting CTF estimates were rejected based on visual inspection, leaving 6,673 videos for processing. Particle auto-picking was performed using the Laplacian-of-Gaussian method in RELION, yielding 1,021,926 particles. These were then extracted from the micrographs with rescaling to 1/8 the full size and underwent three rounds of two-dimensional (2D) classification. The 735,114 particles from the good 2D classes were then re-extracted at 1/4 the full size and imported into cryoSPARC (ref. ^60^) v.3.1.0 for heterogeneous refinement with six volume classes. The reference ribosome volume for this job was generated from PDB 1VY4 coordinates^24^ in EMAN2 (ref. ^61^). Two of the resulting classes corresponded to clean 70S volumes, so these classes were exported back to RELION and pooled (513,480 particles).

To separate out classes with different positions/rotation of the small subunit relative to the large subunit, 50S-focused refinement was first performed on all the particles, followed by 3D classification without alignment into five volume classes. In addition to subtle shifts in the 30S subunit, this step is useful for the removal of particles in minor states like those containing E-site tRNAs only, for example. From there, further pooling and focused classification on A-site and E-site tRNAs (without alignment) were performed, the details of which are summarized in Supplementary Fig. 1a. As all particles that were accepted at this stage contained P-site tRNAs, and the A and E sites appeared to produce more meaningful separation, classification on the P site was not pursued.

For the final structure, all particles in the three classes selected at the 30S rotation stage were extracted at full size and pooled for 50S-focused refinements prior to and in between CTF refinement^62^, Bayesian polishing^63^ and CTF refinement again. This was to take advantage of the high resolution from the well-ordered large subunit and maximize particle number for optimum performance of these jobs, and the resulting post-processed volume was also used for pixel size calibration. Particle classes of interest that had been identified earlier were separated again for final 50S-focused refinements using Python scripting to pull the appropriate subsets from the polished particle metadata.

From the classification procedure, there were three initial A-site classes that were examined individually as well as merged, and with and without further sorting on the E site. Ultimately, variation across maps indicated some residual disorder in fine details of the substrates, with some features alternately improved or worsened in different maps, and there was no single map representing the best set of features across A-site and P-site Met residues or their surroundings (Supplementary Fig. 1b). Taken together, it is clear that we are still somewhat limited by the inherent dynamics of substrates in the active site. Additionally, the line between meaningfully distinctive classes or conformations and the continuous motions of the complex(es) presents a challenge, particularly when particle number starts to become limiting. A merged map of two of the A-site classes was ultimately chosen for modelling, containing 129,455 particles and with a global resolution of the large subunit and tRNAs going to 2.1 Å resolution. A 30S-focused refinement was also performed for modelling purposes, which went to 2.3 Å resolution.

### Pixel size calibration

The pixel size was calibrated in Chimera (ref. ^64^) v.1.16 using the ‘Fit to Map’ function and the high-resolution pooled-particle map against the 50S subunit coordinates from the X-ray crystal structure of PDB 4YBB (ref. ^65^). The best cross-correlation value was obtained at a pixel size of 0.8296 Å. Half-maps for our refined volume were rescaled to the calibrated pixel size in Chimera, and post-processing was performed with these half-maps in RELION to obtain the Fourier shell correlation curve and final resolution estimate (Extended Data Fig. 2).

### Modelling

PDB 7K00 (ref. ^30^) was taken as a starting model. This model contained tRNA^fMet^ in the P site and tRNA^Val^ in the A site, whereas only tRNA^fMet^ was used in the current work, so the A-site tRNA was replaced. Real-space refinement of the coordinates was performed in PHENIX (ref. ^66^; version dev-3051 and v.1.20.1-4487) into the 50S- and 30S-focused maps, and further adjustments to the model were done manually in Coot (ref. ^67^; v.0.9.4 and v.0.9.8.5), mainly around the PTC. Further additions to the model included Mg^2+^ and K^+^ ions, water molecules and spermidine ligands near the tRNA CCA ends, as well as residues 2–7 of r-protein bL27. The linkage between A-site and P-site tRNAs and Met monomers was modelled as an amide linkage to reflect experimental conditions for structure determination. As the 30S subunit was mainly included for model completeness, while the results are largely concerned with the 50S subunit, a map-versus-model Fourier shell correlation was calculated in PHENIX (ref. ^66^) for the 50S coordinates and focused map (Extended Data Fig. 2b). Model refinement statistics for the two subunits are summarized in Supplementary Table 2.

### Composite map

A composite cryo-EM map was calculated from the 2.1 Å 50S map and 2.3 Å 30S map for deposition. The 30S map was aligned to best fit the 30S region of the 50S map and resampled in Chimera. The two maps were set to have volume standard deviation values such that the small subunit (SSU) and large subunit (LSU) are visually balanced in the combined map. Coordinates for the 70S complex are fit to this composite map.

### Molecular dynamics simulations

As described in the Results, the starting point for our MD simulations was the RRM, which involved rigid-body docking of ordered Mg^2+^ ions from PDB 7K00 into preliminary coordinates for the cryo-EM structure reported here, which ultimately included additional ions not present in 7K00. Static structures fail to capture the dynamic interactions between the RNA and ions^68^. Moreover, monovalent cations generally interact with RNA as highly dynamic species, creating a diffusive ion environment, while divalent cations, such as Mg^2+^, form an ion-hydration shell^28,69,70^. Hence, the rigid-body docking of the Mg^2+^ ions from 7K00 described above was considered sufficient for our unbiased and biased MD calculations.

The resulting structure was solvated using the simple point charge water model^71^. K^+^ and Cl^–^ ions corresponding to 0.15 M concentration were added as well as K^+^ counterions to neutralize the system. The final simulation box measured 95 Å along each side and consisted of ~88,000 atoms. The OPLS4 force field^72^ and Desmond MD system (Schrödinger Release 2022-2) as implemented within Schrödinger Suite (release 2022-2) were used in this study. For fMet and all the non-l-α-amino acid monomers, the Force Field Builder (Schrödinger release 2022-2)^72^ was used to parametrize the missing torsions, which it does by fitting the molecular mechanics torsional profiles to those obtained based on quantum mechanics calculations.

The systems were initially minimized and equilibrated with restraints on all solute heavy atoms, followed by production runs with all but the outer 10 Å C1′ and Cα atoms unrestrained. The constant-temperature, constant-pressure (NPT; number of particles *N*, pressure *P*, temperature *T*) ensemble was used with constant temperature at 300 K and Langevin dynamics. The production runs were carried out for 300 ns in triplicate, changing the initial velocity seeds for each run. The conformational analysis, including the distances and angles, was calculated using Schrödinger’s Python API (Schrödinger release 2022-2). The representative pose for the test fMet–Met run (Fig. 2c) was generated using the trajectory r.m.s.d.-based clustering method^73^ as implemented in Maestro. The radial distribution function was generated using Schrödinger’s built-in radial distribution function panel.

### Metadynamics

Desmond^74^ (Schrödinger release 2022-2) was used for the metadynamics runs. The equilibration stage was the same as for the MD runs above, and the metadynamics production runs were carried out in duplicate (starting from different conformations as outlined above) for 100 ns each. The N_α_–C_*sp*2_ distance and the Bürgi–Dunitz angle were used as collective variables. The biasing Gaussian potential (‘hill’) of 0.01 kcal mol^–1^ was used, and a width of 0.15 Å for the N_α_–C_*sp*2_ distance and 2.5° for the Bürgi–Dunitz angle *α*_BD_ were applied. Analysis of the runs was performed with Schrödinger’s Python API (Schrödinger release 2022-2) as well as in-house Python scripts.

### Reporting summary

Further information on research design is available in the Nature Portfolio Reporting Summary linked to this article.

## Online content

Any methods, additional references, Nature Portfolio reporting summaries, source data, extended data, supplementary information, acknowledgements, peer review information; details of author contributions and competing interests; and statements of data and code availability are available at 10.1038/s41557-023-01226-w.

## Supplementary information

Supplementary Information Supplementary Tables 1 and 2 and Figs. 1–6.

Reporting Summary

Supplementary Data 1 CIF file for 8EMM.

## Data Availability

The cryo-EM map and model reported here are deposited at the EM Data Bank and RCSB Protein Data Bank with accession codes EMD-28257 for the 50S-focused map, EMD-28256 for the 30S-focused map, EMD-28255 for the 70S map, EMD-28254 for the composite map and PDB 8EMM for the full 70S complex coordinates aligned to the composite map. The coordinates of the RRM models representing metadynamics global minima are available at Zenodo: 10.5281/zenodo.7730661. The full MD and metadynamics trajectories are too large to upload at Zenodo (>50 GB); however, they are available upon reasonable request. Source data are provided with this paper.

## Source data

Source Data Fig. 2 Data in CSV format for Fig. 2a,b,d.
Source Data Fig. 4 Data in CSV format for the kernel density estimate plots in Fig. 4; each panel and each monomer’s data are in a separate CSV file, named accordingly.
Source Data Fig. 5 Data in CSV format for the FES plots in Fig. 5; each monomer’s data are in a separate CSV file, named accordingly.
Source Data Fig. 6 Data in CSV format for the FES plots in Fig. 6; each monomer’s data are in a separate CSV file, named accordingly.
Source Data Extended Data Fig. 6 Data in CSV format for the trajectory plots in Extended Data Fig. 6; each trial and each monomer’s data are in a separate CSV file, named accordingly.
Source Data Extended Data Fig. 7 Data in CSV format for the scatter plots in Extended Data Fig. 7; each column and each monomer’s data are in a separate CSV file, named accordingly.
Source Data Extended Data Fig. 8 Data in CSV format for the FES plots in Extended Data Fig. 8; each monomer’s data are in a separate CSV file, named accordingly.

## Code availability

Python scripts written using Schrödinger’s API for the analysis of the data from the MD and metadynamics simulations, the metadynamics raw data for the free energy and the collective variables at each time point, and the FES data are available at Zenodo: 10.5281/zenodo.7714401.
