# Supplementary material for: Atomistic simulations of the Escherichia coli ribosome provide selection criteria for translationally active substrates
Source: Nat Chem. 2023 Jun 12;15(7):913–21. doi: 10.1038/s41557-023-01226-w (PMC10322701; doi:10.1038/s41557-023-01226-w)
Supplement: Supplementary file 1 — Supplementary Tables 1 and 2 and Figs. 1–6. [file 41557_2023_1226_MOESM1_ESM.pdf]

Supplementary Table 1. Primers used for tRNA template synthesis (‘mG’ indicates 2’ O-methylguanosine).

tRNA-fMet-C1G_temp F	AATTCCTGCAGTAATACGACTCACTATAGGCGGGGTGGAGCAGCCTGGTAGCTCGTCGGGCTCATA
tRNA-fMet-C1G_amp F	AATTCCTGCAGTAATACGACTCAC
tRNA-fMet-C1G-A_temp R	GGTTGCGGGGGCCGGATTTGAACCGACGACCTTCGGGTTATGAGCCCGACGAGCTA
tRNA-fMet-C1G-A_amp R	GmGCCCCCGCAACC

Supplementary Table 2. Cryo-EM data collection parameters and Refinement and validation statistics.

Cryo-EM data collection, refinement and validation statistics

	50S subunit (EMDB-28257) (PDB 8EMM)	30S subunit (EMD-28256) (PDB 8EMM)
**Data collection and processing**		
Magnification	102,519x	
Voltage (kV)	300	
Electron exposure (e–/Å^2)	40	
Defocus range (μm)	-2 to -0.5	
Pixel size (Å)	0.8296	
Symmetry imposed	C1	
Initial particle images (no.)	1,021,926	
Final particle images (no.)	129,455	
Map resolution (Å)	2.1	2.3
FSC threshold	0.143	0.143
Map resolution range (Å)	1.9 - 3	2.3 - 4
**Refinement**		
Initial model used (PDB code)	7K00	
Model resolution (Å)	2.06	2.4
FSC threshold	0.5	0.5
Model resolution range (Å)	2.06 - 3	2.4 - 4
Map sharpening *B* factor (Å^2)	-13	-25

Model composition		
Non-hydrogen atoms	93656	52079
Protein residues	3184	2409
Ligands	257	95
B factors (Å^2)		
Protein	38.64	35.18
Ligand	23.23	26.46
R.m.s. deviations		
Bond lengths (Å)	0.011	0.006
Bond angles (°)	1.170	0.784
Validation		
MolProbity score	1.23	1.98
Clashscore	2.56	8.82
Poor rotamers (%)	0.50	2.01
Ramachandran plot		
Favored (%)	96.89	95.94
Allowed (%)	3.02	4.02
Disallowed (%)	0.10	0.04

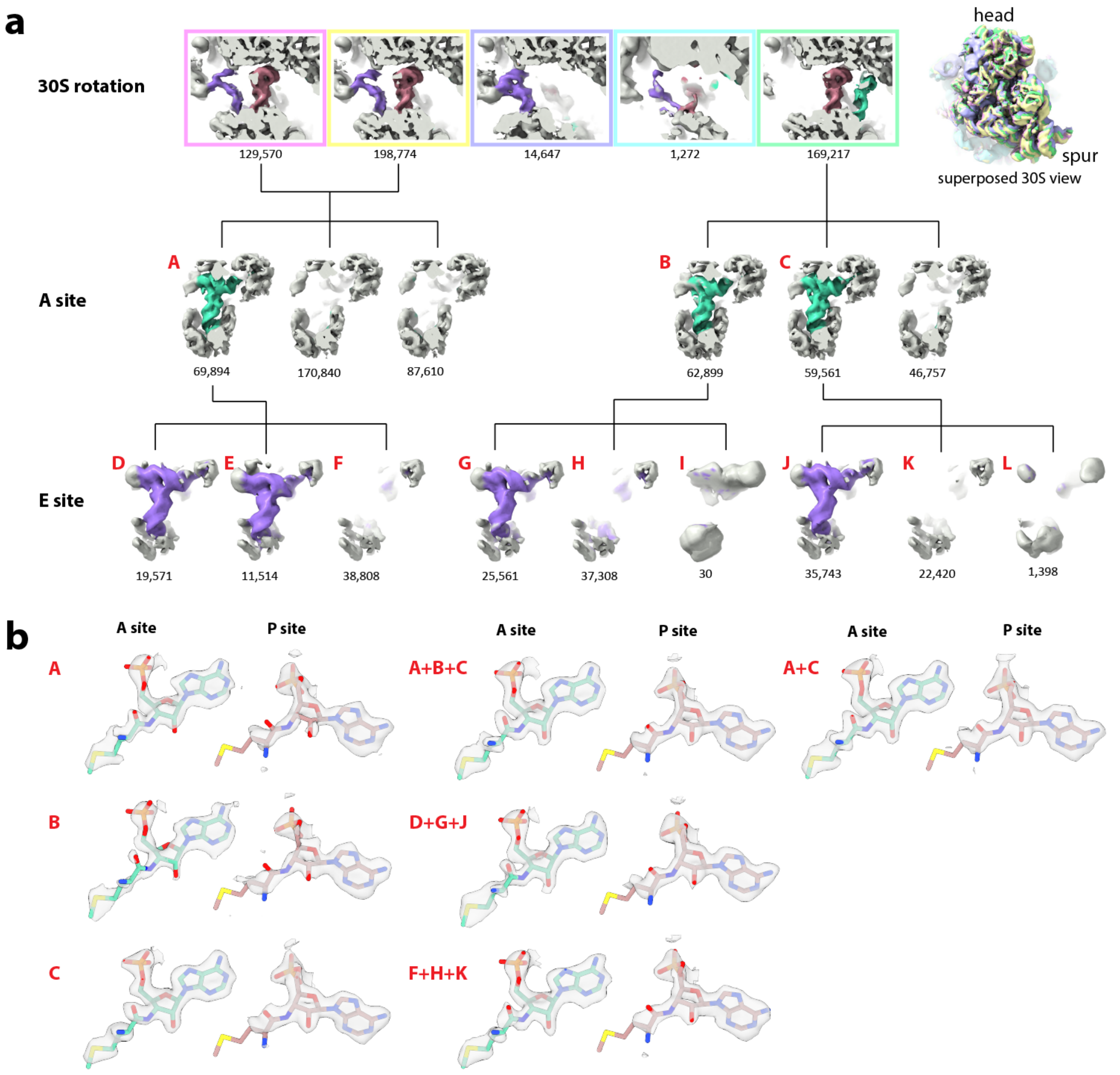

Supplementary Fig. 1. a, Flowchart illustrating classification workflow applied during cryo-EM data processing. Apart from the top right 30S view, density for A-site, P-site, and E-site (or P/E hybrid) tRNAs are shown in green, pink, and purple, respectively, with surrounding ribosomal density in gray. The number of particles in each class is shown beneath its image and all classes with A-site tRNA are identified with an uppercase letter in red. Top row: After an initial consensus 50S-focused refinement, particles were first sorted into 5 classes to discriminate between populations with different relative 30S subunit positions. Pink, yellow, purple, cyan, and green outlines on the zoomed-in cutaway panels are colored to match corresponding superposed whole ribosome maps on the top right, showing subtle rotations of the small subunit. Pink- and yellow-outlined classes were merged based on similarity. Middle row: particles were then classified on the A site. Bottom row: particle classes with A-site tRNA were further classified on the E site. Interestingly, only one of the A-site classes included particles with E-site tRNA in two different positions (classes D and E), however, this did not affect analysis of PTC density. **b,** Close-ups of A- and P-site density for select reconstructions that were compared to be chosen for modeling. While a number of permutations were considered, here we show the initial classes A, B, and C before sorting on the E site, as well as A+B+C and A+C merged. We also show D+G+J merged and F+H+K merged, representing the most homogeneous sets of particles in terms of tRNA occupancy. However, classes A+C were chosen for modeling the PTC. Cryo-EM density is shown in gray.

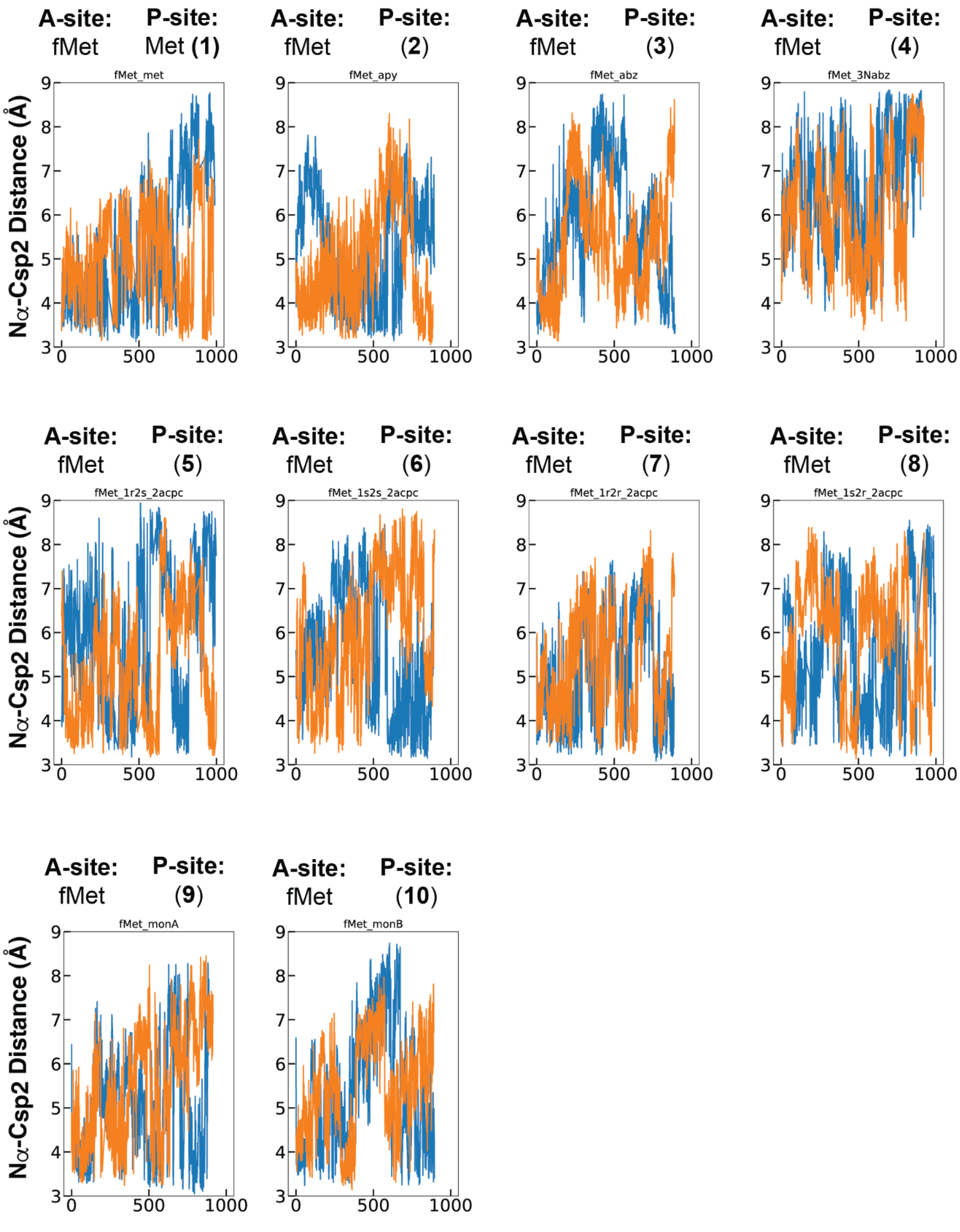

Supplementary Fig. 2. Trajectories of the two metadynamics replicas (blue and orange) showing the fluctuations of the N_{α}-C_{sp2} distance, one of the two collective variables (CVs) used in this work, for all ten monomers evaluated in the ribosomal P-site. The units for the x-axes are the number of frames, 1 frame is 0.1 ns.

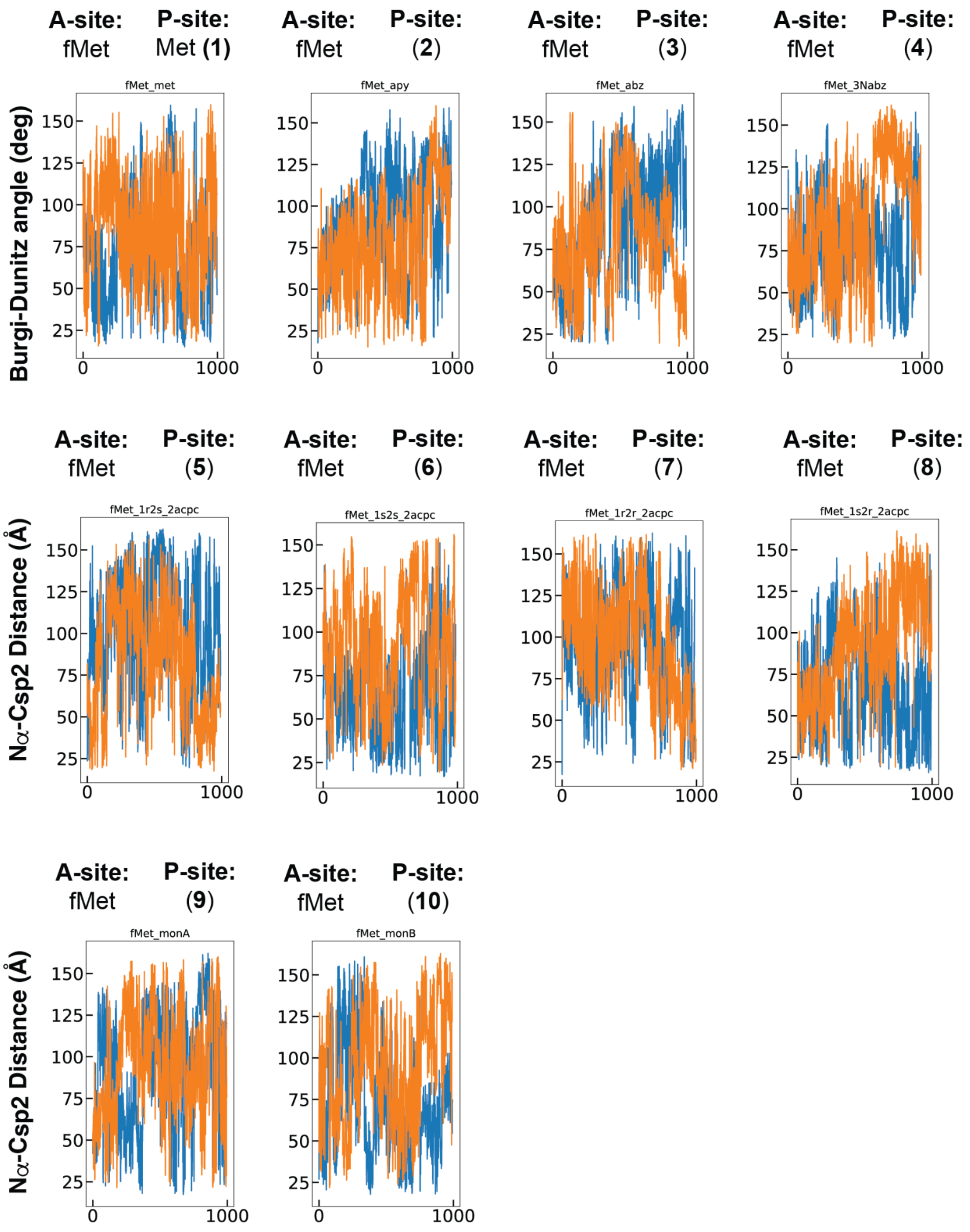

Supplementary Fig. 3. Trajectories of the two metadynamics replicas (blue and orange) showing the fluctuations of the α_{BD} angle, one of the two collective variables (CVs) used in this work, for all ten monomers evaluated in the ribosomal P-site. The units for the x-axes are the number of frames, 1 frame is 0.1 ns.

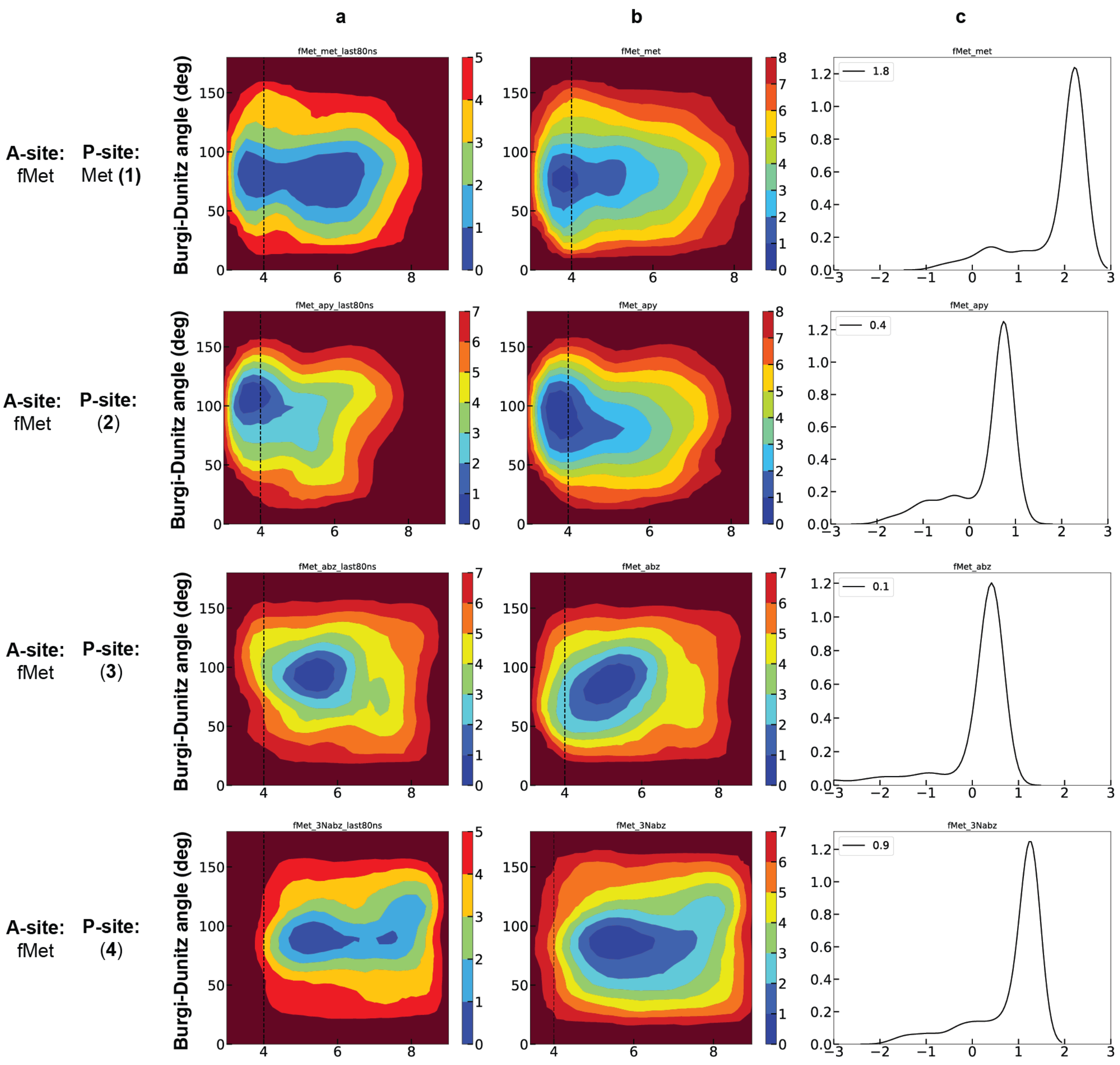

Supplementary Fig. 4: Free energy surfaces (FESs) of **a,** the last 80 ns, **b,** the full 100 ns, and **c,** Kernel Density Estimates (KDEs) of the differences between the 100 ns and the last 80 ns FESs for metadynamics runs of monomers **1-4**. Means are shown in the upper left of the plots in **c.** Data shown represents the aggregate of the duplicate runs.

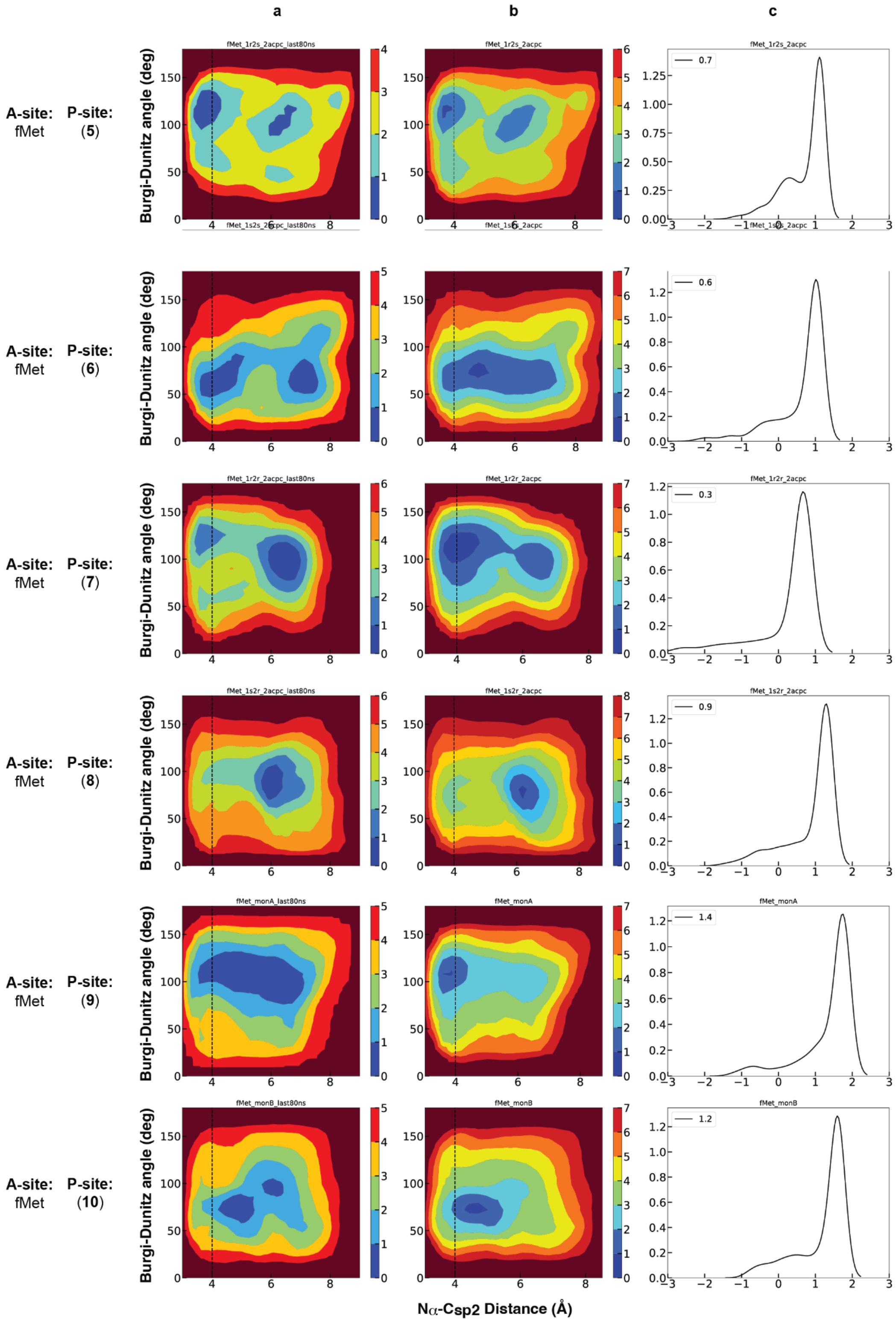

Supplementary Fig. 5: Free energy surfaces (FESs) of **a,** the last 80 ns, **b,** the full 100 ns, and **c,** Kernel Density Estimates (KDEs) of the differences between the 100 ns and the last 80 ns FESs for metadynamics runs of monomers **5-8**. Means are shown in the upper left of the plots in **c.** Data shown represents the aggregate of the duplicate runs.

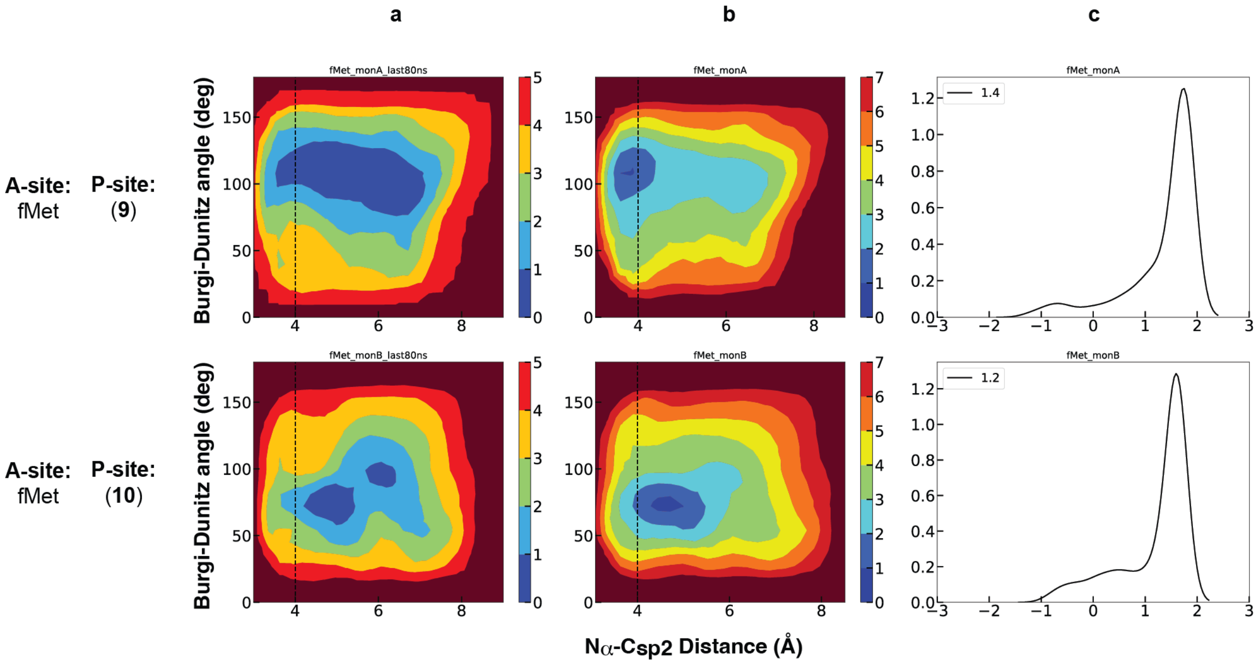

Supplementary Fig. 6: Free energy surfaces (FESs) of **a,** the last 80 ns, **b,** the full 100 ns, and **c,** Kernel Density Estimates (KDEs) of the differences between the 100 ns and the last 80 ns FESs for metadynamics runs of monomers **9** and **10**. Means are shown in the upper left of the plots in **c.** Data shown represents the aggregate of the duplicate runs.
